# Elucidating Events within the Black Box of Enzyme Catalysis in Energy Metabolism: Insights into the Molecular Mechanism of ATP Hydrolysis by F_1_-ATPase

**DOI:** 10.3390/biom13111596

**Published:** 2023-10-30

**Authors:** Sunil Nath

**Affiliations:** 1 Department of Biochemical Engineering and Biotechnology, Indian Institute of Technology Delhi, Hauz Khas, New Delhi 110016, India; sunath@iitd.ac.in or sunil_nath_iit@yahoo.com; 2 Institute of Molecular Psychiatry, Rheinische-Friedrichs-Wilhelm Universität Bonn, D–53127 Bonn, Germany

**Keywords:** rotary ATPases, F_O_F_1_-ATP synthase and F_1_-ATPase, ATP synthesis and hydrolysis, energy metabolism and mitochondria, molecular mechanism, kinetics and conformational change, oxygen exchange and stochastic theory, ligand displacement/substitution and ligand permutation, Boyer’s binding change mechanism of ATP synthesis, Nath’s torsional mechanism of ATP synthesis and two-ion theory of energy coupling, 2-site vs. 3-site models of ATP synthesis/hydrolysis

## Abstract

Oxygen exchange reactions occurring at β-catalytic sites of the F_O_F_1_-ATP synthase/F_1_-ATPase imprint a unique record of molecular events during the catalytic cycle of ATP synthesis/hydrolysis. This work presents a new theory of oxygen exchange and tests it on oxygen exchange data recorded on ATP hydrolysis by mitochondrial F_1_-ATPase (MF_1_). The apparent rate constant of oxygen exchange governing the intermediate Pi–HOH exchange accompanying ATP hydrolysis is determined by kinetic analysis over a ~50,000-fold range of substrate ATP concentration (0.1–5000 μM) and a corresponding ~200-fold range of reaction velocity (3.5–650 [moles of Pi/{moles of F_1_-ATPase}^−1^ s^−1^]). Isotopomer distributions of [^18^O]Pi species containing 0, 1, 2, and 3 labeled oxygen atoms predicted by the theory have been quantified and shown to be in perfect agreement with the experimental distributions over the entire range of medium ATP concentrations without employing adjustable parameters. A novel molecular mechanism of steady-state multisite ATP hydrolysis by the F_1_-ATPase has been proposed. Our results show that steady-state ATP hydrolysis by F_1_-ATPase occurs with all three sites occupied by Mg-nucleotide. The various implications arising from models of energy coupling in ATP synthesis/hydrolysis by the ATP synthase/F_1_-ATPase have been discussed. Current models of ATP hydrolysis by F_1_-ATPase, including those postulated from single-molecule data, are shown to be effectively bisite models that contradict the data. The trisite catalysis formulated by Nath’s torsional mechanism of energy transduction and ATP synthesis/hydrolysis since its first appearance 25 years ago is shown to be in better accord with the experimental record. The total biochemical information on ATP hydrolysis is integrated into a consistent model by the torsional mechanism of ATP synthesis/hydrolysis and shown to elucidate the elementary chemical and mechanical events within the black box of enzyme catalysis in energy metabolism by F_1_-ATPase.

## 1. Introduction

Given the fundamental importance of ATP as the cellular fuel in animals, plants, and eubacteria, the working mechanism of ATP synthesis/hydrolysis in energy metabolism by the F_O_F_1_-ATP synthase/F_1_-ATPase has been studied extensively. Structural studies using X-ray crystallography provided the first snapshots of this vital enzyme in animal mitochondria at high resolution and glimpses of its interacting parts [1,2]. The first crystal structure solved contained bound ATP analog AMP–PNP at one of its catalytic sites (designated as β_TP_), ADP at a second catalytic site (denoted by β_DP_), while a third site (β_E_) was empty of bound nucleotide [1] and seemed to be compatible with prevailing models. However, subsequent X-ray structures have shown that the three β-catalytic sites can contain a variety of nucleotide combinations, including one structure that contains the transition state analog, MgADPAlF_4_^−^, at two sites with the third site filled with MgADP and sulfate (that mimics the cleaved γ-phosphate) [2]. Recent developments in cryo-EM have been exploited by several groups and have provided more detailed structural information on the mitochondrial enzyme [3,4,5,6,7,8]. 

Single-molecule imaging of F_1_-ATPase gave the field spectacular videos of the mechanoenzyme as a rotary device [9,10,11,12,13,14,15]. It was specifically shown that the catalytic dwell in thermophilic F_1_ (TF_1_) is ~2 ms in duration, during which ATP hydrolysis occurs with a half-time of ~1 ms and ends with Pi release [11,15]. Further, bovine mitochondrial F_1_-ATPase (MF_1_) seemed to differ from bacterial F_1_ in that it has a Pi release dwell that occurs prior to the catalytic dwell [14].

In addition to the use of the above biophysical techniques, biochemical approaches continued to be employed to probe the rotary ATPases. Prominent among these was the utility of crosslinking tools, which had provided the first evidence for rotation in the ATPases [16,17]. In order to improve our understanding of the energy dynamics of rotary machines, various energy landscape methods were developed and applied to the ATPases [18,19]. At times, single-molecule and energy landscape approaches yielded insights that were in concurrence with each other. For instance, Frasch and coworkers discovered from single-molecule data collected with an unprecedented signal-to-noise ratio at an ultra-rapid frame rate of 200,000 that rotation in F_1_-ATPase was more complex than previously thought and was accompanied by a series of angular accelerations and decelerations [13] and elastic coupling [10] during the power stroke. Nath reached similar conclusions from his landscape approach that quantified the electrostatic motor torque vs. angle and the angular displacement, angular velocity vs. time relationship over a 120° cycle of the enzyme that incorporated a “wheels-within-wheels” mechanism of twisting [18]. A “wheels-within-wheels” mechanism is now being postulated for the bacterial flagellar motor since it is consistent with recent cryo-EM structures of the “5:2 rotary motors” [20]. However, puzzling dynamical effects were also uncovered by single-molecule recordings. Futai and colleagues found that stochastic fluctuations of catalysis are a general property of the F_1_-ATPase and concluded that understanding it requires “combining studies of steady-state kinetics with single molecule observation” [12]. 

Other rotary and linear molecular motor systems were studied both experimentally and computationally [21,22,23,24,25,26,27,28], and molecular dynamics [24,25] and multiscale simulations [26,27,28] were performed. In addition, electrostatic [29,30] and molecular systems biology/engineering [31,32,33] approaches were developed to understand function. Several reviews on the rapid developments in ATP and cell life [33,34,35,36,37,38] and cell death [39,40,41,42,43,44,45,46,47] were written. A crucial central question was the following: How could time-resolved information [48,49,50,51,52] on elementary events occurring in single catalytic sites of enzymes, such as in the β-subunits of the F_1_-ATPase, be gathered and interpreted? The dynamics of movement of the ε-subunit are incompletely understood and continue to be addressed by structural [8], biochemical [53,54], biophysical [52,55], and systems biology [31] approaches.

**Figure 1:**
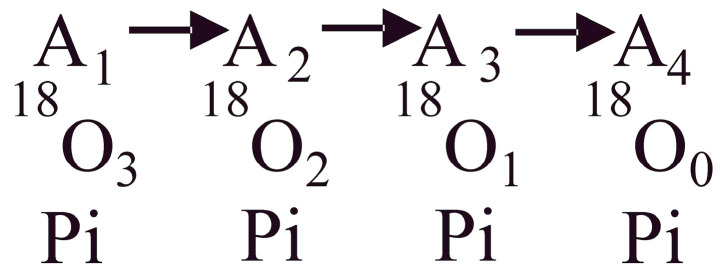
Schematic of the oxygen exchange reactions within a single β-catalytic site in the F_1_ portion of the F_O_F_1_-ATP synthase/F_1_-ATPase. The process starts from an initial state of the system with a known distribution of the ^18^O label in the γ-phosphoryl group of ATP (A_0_; not shown in the schematic). Following terminal bond cleavage of the bound ATP and release of inorganic phosphate (Pi), the system progresses between enzyme states A_1_, A_2_, A_3_, and A_4_ containing respectively 3, 2, 1, and 0 atoms of ^18^O in the released Pi molecule due to intermediate Pi–HOH exchange occurring during ATP hydrolysis, shown by arrows. The ^18^O_3_, ^18^O_2_, ^18^O_1_, and ^18^O_0_ Pi distributions are measured, and the estimated distributions obtained by a stochastic kinetic theory are compared with the experimental distributions.

An interesting methodology by which time-resolved information on catalytic site events can be collated is by monitoring the oxygen exchange reactions [56,57,58]. As is well known, experimental data on the oxygen exchange reactions in the ATP synthesis mode by the F_O_F_1_-ATP synthase are scarce [57]. In particular, the characteristics of the intermediate ATP–HOH exchange, considered as a diagnostic of the oxidative phosphorylation process, were experimentally studied by Nath and coworkers [57]. A stochastic kinetic theory was developed from first principles to support the experiments and to analyze and interpret the experimental data in the synthesis mode. The variables in the concatenated system of differential equations were solved sequentially and shown to lead to a Poisson process as the steady-state solution of the continuous-time Markov chain [57]. However, given that the vast majority of studies have focused on the hydrolysis mode, good data on the exchange reactions occurring during ATP hydrolysis by the F_1_-ATPase, particularly on the intermediate Pi–HOH exchange (Figure 1), needs to be recorded.

The above works have stimulated the development of at least two different theories or mechanisms of ATP synthesis/hydrolysis. Boyer’s binding change mechanism [58,59,60,61] was primarily arrived at based on the action of uncouplers on the exchange reactions [59]. It postulated reversible catalysis and site-site cooperativity and proposed that external energy input is primarily required for the release of bound ATP from a catalytic site but not for its synthesis [59,60,61]. According to a central tenet of the binding change mechanism, steady-state ATP synthesis/hydrolysis by the enzyme occurs with only two of the three β-catalytic sites filled with bound Mg-nucleotides, i.e., by a **bisite** mode of catalysis (**2-site model of ATP synthesis/hydrolysis**) [60,61]. An alternative Nath’s torsional mechanism of energy transduction and ATP synthesis/hydrolysis [18,30,31,32,33,57,62,63,64] considered an irreversible mode of catalysis and asymmetric interactions of the β-catalytic sites with the single copy γ- and ε-subunits of F_1_ and proposed that every elementary step in ATP synthesis requires energy [65,66,67,68,69,70,71,72,73,74]. According to a central tenet of the torsional mechanism, steady-state physiological ATP synthesis/steady-state V_max_ ATP hydrolysis by the F_O_F_1_-ATP synthase/F_1_-ATPase occurs with all three β-catalytic sites filled with bound MgATP or MgADP, i.e., by a **trisite** mode of catalysis (**3-site model of ATP synthesis/hydrolysis**) [32,33]. The results presented here are consistent with oxygen exchange occurring at all three catalytic sites during the process of steady-state V_max_ hydrolysis by F_1_-ATPase at high ATP concentrations, as would be predicted by 3-site models of ATP catalysis.

In this work, the intermediate exchange is defined as occurring when a substrate binds to an enzyme catalytic site, undergoes exchanges, and converts to a product, which is then released (from the site into the medium) and contains atoms incorporated by the exchange. For example, if ATP binds, and the released Pi formed upon hydrolysis contains the exchanged oxygen, then it is an intermediate Pi–HOH exchange. *The oxygen exchange in such an intermediate exchange may take place before, during, or after the reaction*. It should be noted that since oxygen from Pi forms water during ATP synthesis [75], one oxygen atom from medium water gets incorporated into Pi and released during the reverse ATP hydrolysis process (Equation (1)).



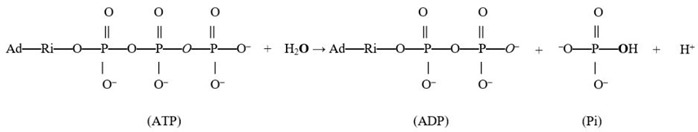

(1)


The presentation of the material is organized in the following way: The experimental methods employed are described in Section 2. Section 3 formulates a new theory of oxygen exchange for ATP hydrolysis by F_1_-ATPase. It is divided into two subsections. The first subsection describes how the stochastic kinetic theory of Mehta et al. (2020) (ref. [57]) on oxygen exchange during ATP synthesis by the F_O_F_1_-ATP synthase is mathematically adapted to treat the oxygen exchange process occurring during ATP hydrolysis by the F_1_-ATPase. In particular, kinetic equations for the *overall* extent of oxygen exchange during ATP hydrolysis by the enzyme are derived in this subsection (Section 3.1). Note that the relevant equations in the two modes, i.e., synthesis and hydrolysis, although possessing a similar form, are not exactly identical to each other [57], Section 3.1. Section 3.2 extends the kinetic theory for the calculation of *isotopomer distributions* of various [^18^O] species – containing 0, 1, 2, or 3 labeled oxygens—in the released Pi molecule. The derived expressions show an underlying mathematical similarity to those of stochastic Poisson-type theory. The final equations for predicting the population distributions (Equations (13)–(16) in Section 3.2) are shown to be obtained by direct integration of the kinetic rate equations (Equation (12)).

The results of applying the kinetic theory developed in Section 3 as applied to our oxygen exchange data are shown in Section 4. The apparent rate constant of oxygen exchange during ATP hydrolysis by F_1_-ATPase is determined over a 50,000-fold concentration range of substrate ATP (Section 4.3). The experimental distributions of [^18^O]Pi species over the entire ATP concentration range are compared with the theoretical population distributions in Section 4.4 using Equations (13)–(16) of Section 3.2. Comparison of experiment with the predictions of (i) Boyer’s binding change mechanism, which postulates a single site/route of water exchange (n=1), and (ii) Nath’s torsional mechanism of energy transduction and ATP synthesis/hydrolysis that invokes multiple sites of oxygen exchange occurring on the MgATP/MgADP.Pi bound at β-subunit interfaces with the α-subunits of F_1_ (n>1, with n=2 or n=3, depending on [ATP] concentrations in the hydrolysis mode).

The mechanism of oxygen exchange and mechanistic implications of the results are discussed in Section 5.1, Section 5.2, Section 5.3, Section 5.4 and Section 5.5. A detailed model for multisite V_max_ hydrolysis by F_1_-ATPase that results from these considerations is presented in Section 5.6. Biological implications arising, such as the order of product release steps during ATP hydrolysis and its consonance with structural data (Section 5.7), the identity of site 2 in the high-resolution X-ray structures (Section 5.8), the angle at which the elementary chemical events of binding, hydrolysis, Pi, and ADP release occur during the ATPase catalytic cycle (Section 5.9) are discussed. The consistency of the proposed mechanism with the results of Weber, Senior, and colleagues by direct measurement of catalytic site nucleotide occupancies using optical probes engineered into β-catalytic sites of F_1_-ATPase and relationship with other models of ATP hydrolysis, including those based on single-molecule observations, is detailed in Section 5.10. The main conclusions arising from our studies of oxygen exchange by F_1_-ATPase are stated point-wise in Section 6.

## 2. Materials and Methods

### 2.1. Materials

MF_1_-ATPase was isolated using the method of Penefsky [76] and stored as a 50% (NH_4_)_2_SO_4_ suspension at 4 °C, pH 8.0 in a medium containing 125 mM sucrose, 25 mM Tris-HCl (Merck, Darmstadt, Germany), 1 mM EDTA (Sigma-Aldrich, St. Louis, MO, USA), and 2 mM ATP (Merck, Darmstadt, Germany). Then, 0.1 mL aliquots of the suspension were pelleted, and the pellet was washed two times in the above buffer without ATP. The pellet was dissolved at room temperature by adding 0.1 mL of a solution of 50 mM Tris-sulfate, 50 mM K_2_SO_4_, and 1 mM EDTA at pH 8.0, followed by centrifugation through a Sephadex G-50 column pre-equilibrated with the buffer solution. Protein concentration was measured by the method of Lowry, and the concentration was converted to dry weight basis as given previously [76]. The specific activity of the ATPase was measured at 105 µmol min^−1^ mg^−1^ at pH 7.4, 30 °C. The variation of the ATPase-specific activity over time was measured within 10% of the above value.

### 2.2. ATP Hydrolysis by F_1_-ATPase

The ATPase reaction rate was measured as follows. The 3 mL reaction mixture contained ADP and F_1_-ATPase at the desired concentration, 5 mM magnesium acetate, 0.2 mM potassium phosphoenolpyruvate (Millipore Sigma, Burlington, MA, USA), and 0.05–0.5 mg/mL of pyruvate kinase (Thermo Fisher Scientific, Waltham, MA, USA) in 30 mM Tris-acetate, 30 mM potassium acetate buffer, pH 7.4 at 30 °C. The addition of pyruvate kinase to the reaction mixtures was made before the addition of F_1_-ATPase, and sufficient time was given for the conversion of almost all the ADP present to be converted to ATP. At various times after the addition of the F_1_-ATPase, a 0.5 mL sample was withdrawn and added to a cuvette containing 0.27 mL buffer, 0.03 mL of 3 mg mL^−1^ of NADH (Merck, Darmstadt, Germany), and 0.2 mL of 50 mM sodium EDTA at pH 8.0, which quenched the reaction. A 0.03 mL aliquot containing catalytic amounts of lactate dehydrogenase was added after the cuvette was inserted into the spectrophotometer. The amount of pyruvate formed was determined by the decrease in absorbance at 340 nm owing to NADH oxidation, and the rate of the ATPase reaction was calculated. The quantity of pyruvate produced during the conversion of ADP to ATP before the addition of F_1_-ATPase was subtracted from the total amount of pyruvate present.

### 2.3. Oxygen Exchange

Oxygen exchange experiments were performed at pH 7.4 and 30 °C in 30 mM Tris-acetate and 30 mM potassium acetate buffer. The ATPase reactions were started by the addition of varying volumes of F_1_ and continued for sufficient time to generate 50–100 nmol per ml of sample. The final sample volume measured was 2.5 mL. The F_1_-ATPase concentration ranged from 70 nM to 10 nM as ATP concentration was increased. The concentration of ATP is the final total concentration of ATP after the reaction is quenched. The quenching was done by vortexing with 2 mL of cold chloroform (Merck, Darmstadt, Germany), and the samples were placed on ice. For each sample, an aliquot of 1 mL was withdrawn for analysis by gas chromatography coupled with mass spectrometry (GC-MS), and the distribution of ^18^O in the Pi was determined as described below. In another set of experiments, 0.5 mL reaction mixtures containing 5 mM magnesium acetate and varying amounts of [^18^O]ATP in the Tris-acetate buffer (Merck, Darmstadt, Germany) at 30 °C were made. F_1_-ATPase of 5–6 mg mL^−1^ concentration was diluted 10-fold, and 5 µL was added to 50 µL buffer at 30 °C. The reaction was initiated by adding 0.45 mL of reaction mixture to the ATPase. The reaction was quenched by the addition of 0.5 mL of cold chloroform after approximately 100 nmol Pi had been produced.

### 2.4. Separation of Pi

The Pi formed by the F_1_-ATPase was isolated by taking aliquots of quenched reaction mixtures adjusted to 1 mL with H_2_O. Then, 0.5 mL phenol was added and centrifuged to remove the protein with the phenol. The phenol layer was washed with 1 mL of H_2_O which was added to the original aqueous layer. The phenol present in the combined aqueous layers was extracted into 2 mL of 1:1 isobutenol:benzene by volume. Molybdate was added, the phosphomolybdate extracted with isobutenol:benzene (Sigma-Aldrich, St. Louis, MO, USA), and the Pi was extracted into an alkaline H_2_O solution. The Pi was separated from the molybdate and converted to H_3_PO_4_ using column chromatography techniques [77]. The H_3_PO_4_ was lyophilized, converted to triethylphosphate with diazoethane, and analyzed for [^18^O]Pi species by GC-MS (Agilent Technologies, Santa Clara, CA, USA).

### 2.5. Determination of ^18^O in Pi

Lyophilized samples of H_3_PO_4_ were derivatized with diazoethane in ethane [77], the excess organic was evaporated, and the resulting triethylphosphate dissolved in dichloromethane (Merck, Darmstadt, Germany) at a concentration of 1 nmol µL^−1^. Aliquots containing about 1 nmol of sample were analyzed for ^18^O with a Hewlett-Packard GC-MS system. With the injection port at 250 °C, isothermal elution at 160 °C, and a gas flow rate of 30 mL min^−1^, the triethylphosphate eluted in around 60 s. The diethylphosphate ion fragment of m/z=155 and its ^18^O isotopomer cousins characterized by m/z ratios of 157, 159, 161, and 163 were analyzed by selective ion monitoring. These species were designated as ^18^O_0_, ^18^O_1_, ^18^O_2_, as well as ^18^O_3_ and ^18^O_4_ Pi, respectively. 

### 2.6. Calculations from Oxygen Exchange Measurements

The data were analyzed using the theory developed and customized for ATPase reactions as described in Section 3.

## 3. Theory

### 3.1. Kinetic Analysis of the Overall Extent of Oxygen Exchange during ATP Hydrolysis

We now adapt the kinetic theory of Nath and coworkers [57] derived for ATP synthesis by F_O_F_1_ to ATP hydrolysis by F_1_-ATPase. We delineate the concentration of the exchanging species (Pi or the γ-phosphoryl of ATP) at any time t as C and its initial concentration as $C_{i}$. We denote X as the fractional extent of ^18^O incorporated into the Pi released from the catalytic site into the medium after time t, and $X_{i}$ as the fraction of ^18^O in Pi at time t=0. t is, therefore, the time for which oxygen exchange takes place from a *single* catalytic site during ATP hydrolysis by F_1_-ATPase, and k is the apparent rate constant of the exchange. For ^18^O exchange from Pi or ATP into water we have

(2) $$-\frac{dC}{dt}=kC$$

Writing C on either side of Equation (2) as $C_{i}\left(1-X\right)$, we obtain the first-order ordinary differential equation for the fractional extent as

(3) $$\frac{dX}{dt}=k\left(1-X\right)$$

Separating variables and integrating from time t=0 to t, we obtain

(4) −ln(1−X)=kt+C 

Upon inserting the initial condition $X=X_{i}$ at t=0 and determining C in Equation (4), we obtain the solution

(5) $$ln\left[\frac{1-X_{i}}{1-X}\right]=kt$$

Following the path of a single ^18^O atom and making use of Equation (5), we can analyze all the possibilities that may arise for the intermediate P_i_–HOH exchange process accompanying ATP hydrolysis. The initial condition for $X_{i}$ can also be accordingly derived. An interesting case is that of the ^18^O label initially present on the γ-phosphoryl group of ATP, and the hydrolysis of ATP occurring first on the enzyme catalytic site, followed by the exchange between the ^18^O oxygens of the bound P_i_ formed after the hydrolytic cleavage reaction and the ^16^O of medium HOH (or vice-versa, if initially the ^18^O label was present in water as H^18^OH), followed by the release of P_i_ from the site into the medium. For this case, the initial ^18^O fractional oxygen of P_i_ (i.e., the fraction at t=0 before the oxygen exchange occurs) has the value $X_{i}=0.25$. This is because in ATP synthesis, oxygen from Pi forms water, while in the reverse hydrolysis mode, the oxygen atom from ^18^O-labeled water is incorporated as one of the four oxygen atoms contained in the Pi molecule [75], as shown in Equation (1). Substituting this initial condition in Equation (5) yields the final result

(6) $$ln\left[\frac{0.75}{1-X}\right]=kt$$

Another interesting case is that of the oxygen exchange between the ^18^O of the labeled γ-PO_3_ group of ATP with water (or vice-versa, if H^18^OH is used) occurring first, followed by the hydrolysis of ATP and thereafter the release of the P_i_ formed into the medium. In this case, $X_{i}$ in Equation (5) is zero, which yields an X before hydrolysis, $X_{Ai}$ of [1−exp(−kt)]. However, only three-fourths of the exchange takes place by this pathway, with the remaining one-fourths arising from the hydrolytic pathway because one water oxygen atom is incorporated into each P_i_ by the hydrolysis reaction itself [75], as shown in Equation (1). Hence, for this case, the fractional ^18^O incorporated in P_i_ after hydrolysis is given by the weighted sum [(3/4)$\;X_{Ai}$ + (1/4)], which again leads to the same final result when expressed in logarithmic form as given by Equation (6). Hence, Equation (6) is quite general and is valid irrespective of the order of the elementary steps during the oxygen exchange and ATP hydrolysis by F_1_-ATPase. 

### 3.2. Stochastic Kinetic Theory for Calculation of Isotopomer Distributions of Various [^18^O] Species in Released Pi during ATP Hydrolysis 

The oxygen exchange reactions taking place within a single enzyme catalytic site during ATP hydrolysis are represented by the overall kinetic scheme of Figure 1. A_1_, A_2_, A_3_, and A_4_ represent the progression of the enzyme system with defined concentrations respectively of [^18^O_3_], [^18^O_2_], [^18^O_1_], and [^18^O_0_] species in Pi for a specified initial distribution, A_0_ of ^18^O in the γ-PO_3_ group of substrate ATP, with $A_{10},\;A_{20},A_{30},A_{40}$ standing for the percentage of the labels with the 3, 2, 1, and 0 labels in the initial distribution. Let $A_{n}$ represent states 1 to n of the enzymatic system containing a certain distribution of the ^18^O with 3, 2, 1, and 0 labels that progressively change with time due to oxygen exchange. 

Following several authors [78,79,80], a stochastic differential equation-based approach was developed for the ATP synthesis/hydrolysis to determine $P_{n}\left(t\right)$, the probability that the system is in state $A_{n}$ at time t for rate constants $k_{n}$ that generally depend on $A_{n}$. This led to the differential equation for n>0

(7) $$\frac{dP_{n}\left(t\right)}{dt}=P_{n-1}\left(t\right)k_{n-1}-P_{n}\left(t\right)k_{n}$$

and for n=0 of

(8) $$\frac{dP_{0}\left(t\right)}{dt}=-P_{0}\left(t\right)k_{0}$$

Computer simulation of the system with different values of $k_{n}$ for n=1 to 4 did not offer any meaningful insights even with the three extra parameters. We then noticed the important point that the differential equations of the general stochastic model (Equations (7) and (8)) reduce to a *Poisson process* if $k_{n}=k$ for all n≥0. Incorporating this restriction on our system of exchange reactions leads to a final differential equation model for the Poisson process that also provides novel physical insights. We therefore have

(9) $$\frac{dP_{n}\left(t\right)}{dt}=P_{n-1}\left(t\right)k-P_{n}\left(t\right)k$$

And

(10) $$\frac{dP_{0}\left(t\right)}{dt}=-P_{0}\left(t\right)k$$

The chemical kinetics of the series reaction system for a population of enzyme molecules (Equation (11)) can be directly written as a set of ordinary differential equations using mass action principles (Equation (12)):

(11) $$A_{1}\to A_{2}\to A_{3}\to A_{4}$$

(12) $$\frac{dA_{1}}{dt}=-kA_{1}\;\frac{dA_{2}}{dt}=kA_{1}-kA_{2}\;\frac{dA_{3}}{dt}=kA_{2}-kA_{3}\;\frac{dA_{4}}{dt}=kA_{3}$$

Integrating and solving the set of ordinary differential equations (Equation (12)) recursively [57,78,79,80] with the initial conditions $A_{10},\;A_{20},A_{30},A_{40}$ (%) for labels with three to zero oxygen atoms in the γ-phosphoryl of ATP leads to the solution

(13) $$A_{1}=\left[18O_{3}\right]=A_{10}e^{-kt}$$

(14) $$A_{2}=[18O_{2}]=A_{10}kte^{-kt}+A_{20}e^{-kt}$$

(15) $$A_{3}=[18O_{1}]=\frac{A_{10}k^{2}t^{2}e^{-kt}}{2}+A_{20}kte^{-kt}+A_{30}e^{-kt}$$

And

(16) $$A_{4}=[18O_{0}]\%=100-\left[18O_{3}\right]\%-\left[18O_{2}\right]\%-\left[18O_{1}\right]\;\%$$

The similarity in the form of the above chemical kinetics equations with the mathematical equations of a Poisson-type model of the process is useful because the exchange reactions satisfy the requirement that the *average* time between oxygen exchange events is set by a particular experimental variable (e.g., substrate ATP concentration) despite the exchange events themselves being *stochastic*, with the exact timing of the events possessing a random nature. The oxygen exchange events are also independent of each other, and the occurrence of one exchange event does not influence the probability of the occurrence of a second exchange event. Moreover, two or more exchange events do not occur simultaneously at a single catalytic site of the enzyme, and the average rate of oxygen exchange events per unit time in the population of enzyme molecules is constant. 

## 4. Results

This study attempts to analyze and quantify the overall extent of the intermediate Pi–HOH exchange and predict the resulting Pi isotopomer distributions as a function of time during ATP hydrolysis by mitochondrial F_1_-ATPase. 

### 4.1. Overall Extent of Oxygen Exchange by F_1_-ATPase as a Function of ATP Concentration

**Figure 2:**
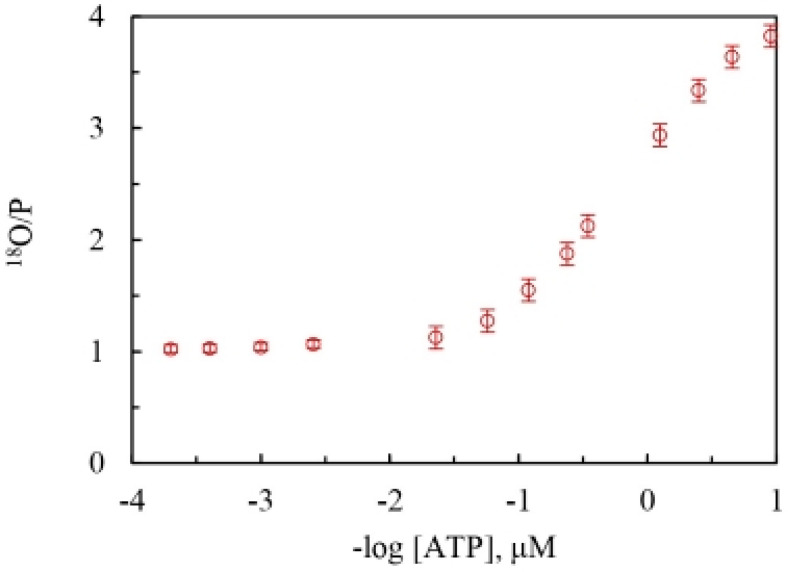
Experimentally-determined average number of ^18^O water oxygen atoms present in each molecule of Pi released (^18^O/P, mean ± SD, *n* = 4) during ATP hydrolysis by mitochondrial F_1_-ATPase (MF_1_-ATPase) over a ~50,000-fold range of concentrations of medium ATP (~0.1–5000 μM).

Figure 2 plots the mitochondrial adenosine triphosphatase data of the overall extent of oxygen exchange as a function of ATP concentration. It shows the variation of the ^18^O/P ratio with changing ATP concentration on a semi-log scale and estimates the average number of ^18^O water oxygens incorporated into Pi by intermediate Pi–HOH exchange occurring during ATP hydrolysis by the F_1_-ATPase. At saturating ATP concentrations, ~0.01 oxygens are transferred to Pi by exchange, while, on the other hand, at low ATP concentrations, ~0.99 fraction of the oxygens in Pi released from the enzyme site into the medium are derived from the ^18^O in water or the ^18^O in the γ-phosphoryl group of ATP, depending on where the label is situated.

### 4.2. Fractional Extent of Oxygen Exchange as a Function of Time during ATP Hydrolysis by F_1_-ATPase

**Figure 3:**
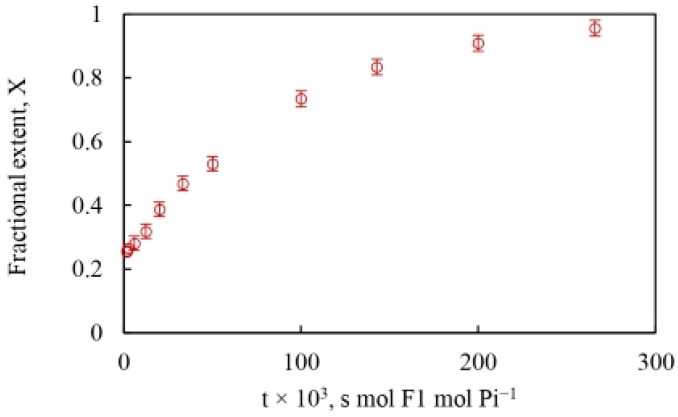
Fractional extent, X of ^18^O incorporated into each Pi released from the enzyme catalytic site into the medium during ATP hydrolysis by MF_1_-ATPase (mean ± SD, *n* = 4) based on the stochastic kinetic theory in Section 2. The *X*-axis plots time, t from the reciprocal of the steady state reaction velocity v [moles of Pi/{moles of F_1_-ATPase}^−1^ s^−1^].

The time scale of the oxygen exchange measurements is determined by the reciprocal of the steady-state velocity during ATP hydrolysis that enables the analysis of the modulation of the fractional extent of oxygen exchange, $X=\frac{18O}{P}/4$ by the ATP concentration, and allows calculation of a time-resolved X at each velocity corresponding to a particular medium ATP concentration. This is shown in Figure 3. Equation (6) also reveals that the rate of oxygen exchange, given by the product of the apparent rate constant of exchange and the time of exchange, can be readily determined at each value of X.

Figure 3 and Equation (6)—which make no specific assumptions regarding mechanism—show that any difference in the rate of oxygen exchange can either be (i) due to a difference in the value of the apparent rate constant, k, of the exchange reaction itself [58,60], or (ii) due to a difference in the lifetime of the exchanging intermediates, i.e., due to the time available for exchange, t [32,33,57]. 

### 4.3. Evaluation of the Apparent Rate Constant of Oxygen Exchange during ATP Hydrolysis by F_1_-ATPase

**Figure 4:**
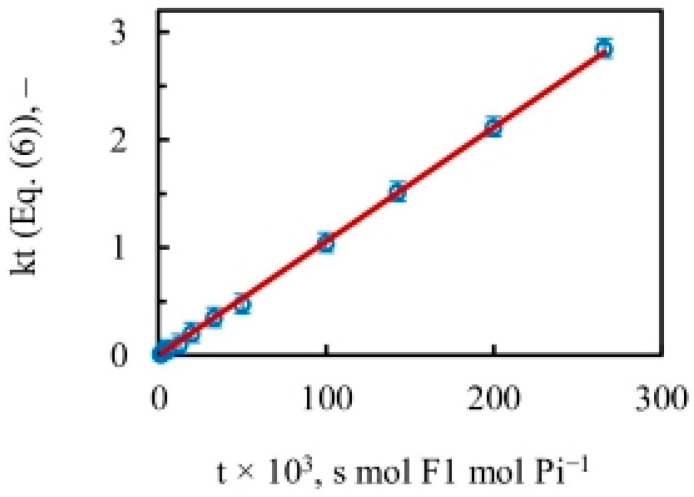
Amount of intermediate Pi–HOH exchange during ATP hydrolysis by MF_1_-ATPase (Equation (6)) as a function of time (mean ± SD, *n* = 4). The slope of the almost-perfect straight line over five decades of medium ATP concentration ($R^{2}=0.999$) gave a constant value for the apparent rate constant of oxygen exchange (k) of 10.5 ± 0.1 s^−1^. The *X*-axis is a measure of time, t plotted as the reciprocal of the steady state reaction velocity v [moles of Pi/{moles of F_1_-ATPase}^−1^ s^−1^].

The results plotted in Figure 4 using Figure 3 and Equation (6) yield a close-to-perfect straight line with *R*^2^ = 0.999. The data thus reveal a *constant* slope with a value of the apparent rate constant of oxygen exchange (k) of 10.5 ± 0.1 s^−1^ over the *entire* substrate concentration range, i.e., over five decades of ATP concentration during steady-state ATP hydrolysis by mitochondrial F_1_-ATPase. It ought to be emphasized that, contrary to expectations from a Boyerean model [58], no variation (increase) in the value of the apparent rate constant of oxygen exchange was found as medium ATP concentration was increased from ~0.1 µM–5 mM, i.e., across a 50,000-fold concentration range of [ATP] (Figure 4).

### 4.4. Quantification of the Distributions of [^18^O]Pi Species at Various ATP Concentrations during ATP Hydrolysis by F_1_-ATPase

**Figure 5:**
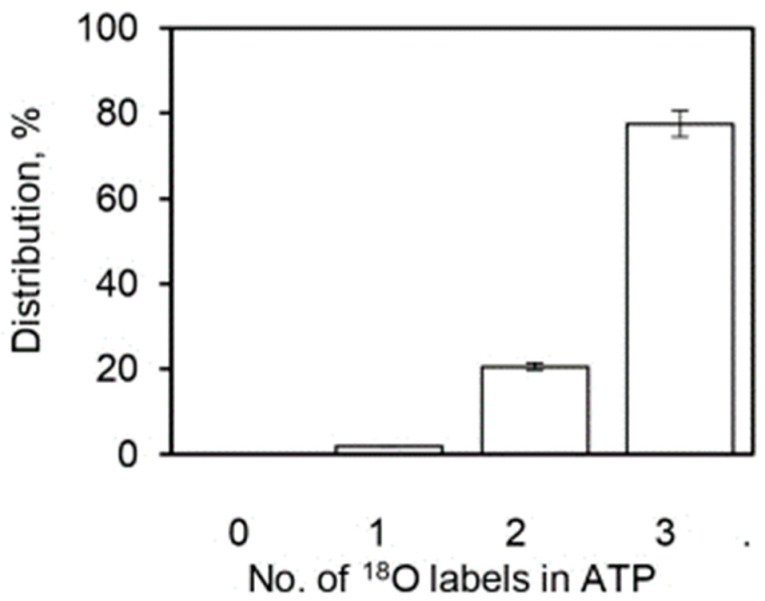
Initial distribution of the ^18^O oxygen atoms (%) in the γ-PO_3_ group of substrate ATP (*n* = 3). The *X*-axis represents the number of labeled oxygens (0, 1, 2, 3) in the starting distribution.

Section 4.1, Section 4.2 and Section 4.3 quantified the average extent of the ^18^O/P ratios arising from the Pi–HOH exchange during ATP hydrolysis by the F_1_-ATPase. The experiments also measured the pattern of distribution of the various [^18^O]Pi species over five decades of ATP concentrations by mass spectrometry (Section 2). In other words, detailed information on the number of ^18^O atoms (0, 1, 2, 3) transferred to each released Pi molecule from the γ-phosphoryl group of ATP upon intermediate Pi–HOH exchange occurring by ligand permutation about the phosphorus center [81] is available. The experimental and theoretically-predicted distributions of such [^18^O]Pi species containing 0, 1, 2, or 3 ^18^O atoms as a percentage of the total have been compared using Equations (13)–(16) as a function of medium ATP concentration, given the initial distribution of ^18^O in the γ–PO_3_ group of ATP shown in Figure 5. 

The comparison of experimental and theoretical [^18^O]Pi distributions at each medium ATP concentration or time point generated a vast amount of data, and we sought a cleaner and short-form way to represent the results. A C^++^ computer program was written that collated data at each temporal point for [^18^O]P_3_, [^18^O]P_2_, [^18^O]P_1_, and [^18^O]P_0_, i.e., with [^18^O_3_], [^18^O_2_], [^18^O_1_], and [^18^O_0_] species in the released Pi after oxygen exchange by F_1_-ATPase (Supplementary Materials) for the starting distribution in Figure 5. The code used Equations (13)–(16) of the stochastic kinetic theory to model the predicted distributions at each time point. It should be noted that since the distributions in Equations (13)–(16) are written in terms of kt, and both t and k are available from the experimental data (Figure 4), *no fitted or adjustable parameters* were employed in arriving at the predicted [^18^O]Pi distributions.

**Figure 6:**
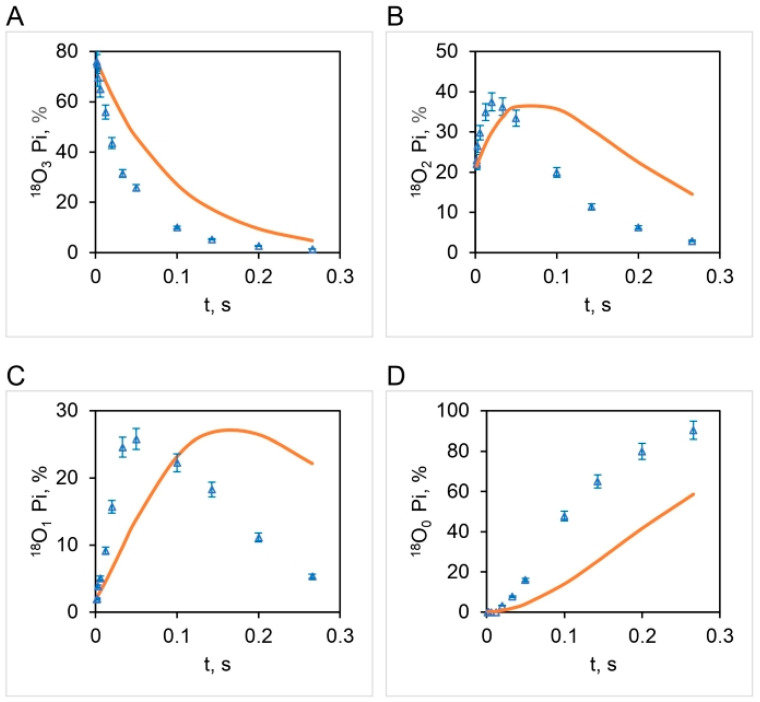
Experimental (open triangles: mean ± SD, *n* = 3) and theoretical distributions (bold curve) of ^18^O (%) in released Pi as a function of time, t, during ATP hydrolysis by mitochondrial F_1_-ATPase for a single site/point of oxygen exchange/water entry. The starting distribution of ^18^O in the γ-phosphoryl group of ATP is given in Figure 5. Pi with three labels, ^18^O_3_ (**A**), two labels, ^18^O_2_ (**B**), one label, ^18^O_1_ (**C**), and zero label of ^18^O in Pi, ^18^O_0_ (**D**).

**Figure 7:**
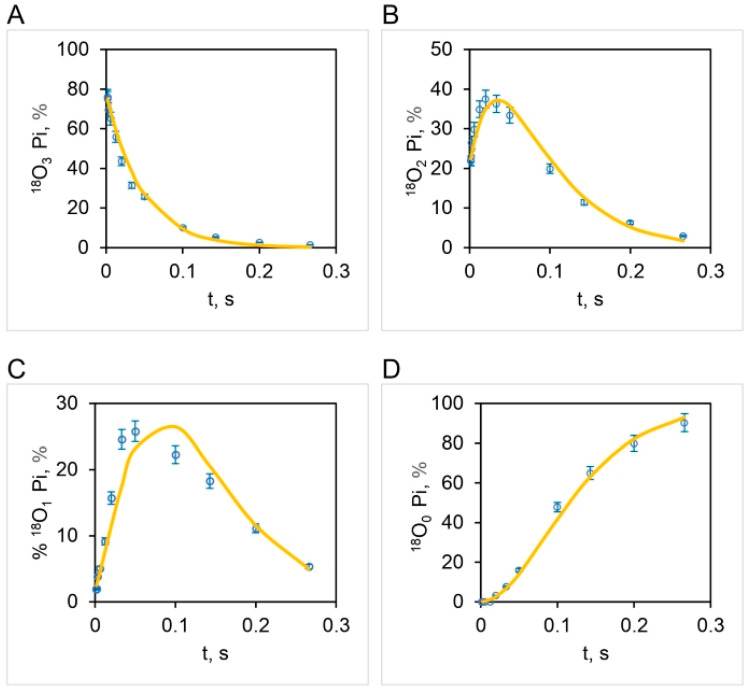
Comparison of experimental (open circles: mean ± SD, *n* = 3) and theoretical isotopomer distributions (bold curve) of ^18^O (%) in released Pi as a function of time, t, during ATP hydrolysis by mitochondrial F_1_-ATPase. The bold curves simulate oxygen exchange over the entire ~50,000-fold range of ATP concentrations (~0.1–5000 μM) for two sites/points of oxygen exchange/water entry, given a starting distribution of ^18^O in the γ-phosphoryl group of ATP of Figure 5. Triple-labeled Pi, ^18^O_3_ (**A**), double-labeled Pi, ^18^O_2_ (**B**), single-labeled Pi, ^18^O_1_ (**C**), and Pi with no label, ^18^O_0_ (**D**).

Boyer’s binding change mechanism of ATP synthesis/hydrolysis involves a single site or mode of entry of water oxygen [59,60]. This key assumption of the mechanism has not been subjected to scientific scrutiny previously. Figure 6 illustrates the results of such a test. The results show that an oxygen exchange model with a single site of entry of ^18^O water and k= 10.5 s^−1^ (Figure 4) does not properly predict the observed distributions of the four [^18^O]Pi species at any substrate ATP concentration (Figure 6). The torsional mechanism of ATP synthesis/hydrolysis, on the other hand, predicts multiple (>1) sites/points of entry of water oxygen [32]. This is the number of catalytic sites at which ^18^O-labeled water can interact with bound ATP or bound ADP.Pi at the same time. A model in which oxygen exchange occurs at the constant value of the apparent rate constant, k, of 10.5 s^−1^ governing the exchange process under the experimental hydrolysis conditions at each ATP concentration at two catalytic sites, essentially doubling the time of exchange, t, in Figure 3 and Figure 4—from what is anticipated to occur at a single catalytic site during a given time—is shown in Figure 7.

**Figure 8:**
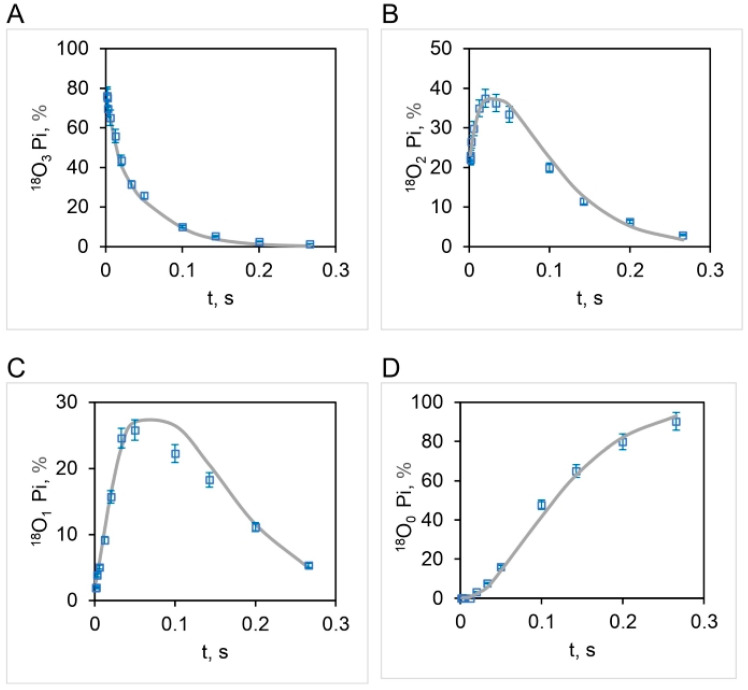
Comparison of experimental (open squares: mean ± SD, *n* = 3) and theoretical isotopomer distributions (bold curve) of ^18^O (%) in released Pi as a function of time, t, over a ~50,000-fold range of ATP concentrations (0.11–5000 μM) during ATP hydrolysis by mitochondrial F_1_-ATPase. The bold curves depict the oxygen exchange based on the stochastic kinetic theory developed in Section 3 for *three* sites/points of oxygen exchange/water entry at short times (0–33 ms) corresponding to high substrate ATP concentrations (4.2–5000 μM), and *two* sites/points of exchange/water entry at long times (33–266 ms), i.e. at low substrate ATP concentrations (0.11–4.2 μM) for an initial distribution of ^18^O in the γ-phosphoryl group of ATP given in Figure 5. Tri-labeled Pi, ^18^O_3_ (**A**), bi-labeled Pi, ^18^O_2_ (**B**), uni-labeled Pi, ^18^O_1_ (**C**), and Pi with no label, ^18^O_0_ (**D**).

**Figure 9:**
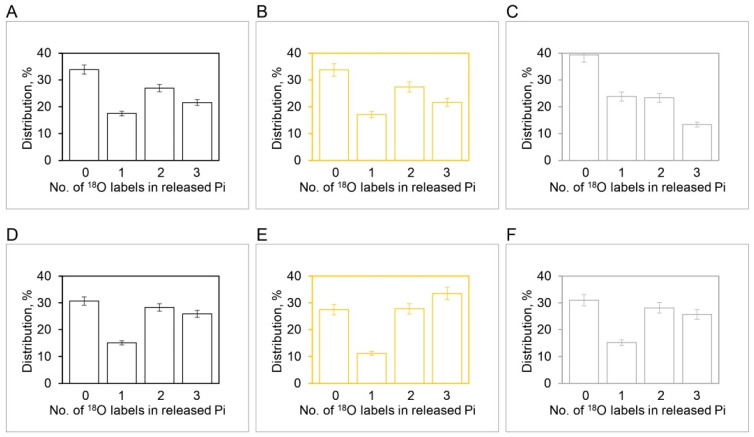
Experimental and theoretical [^18^O]Pi distributions (%) during ATP hydrolysis by MF_1_ at 3 µM ATP (Average ^18^O/P = 2.10: (**A**–**C**)) and 5 µM ATP (Average ^18^O/P = 1.85: (**D**–**F**)) based on experiments in the region of intermediate ATP concentrations (mean ± SD, *n* = 6). Experimental distributions at 3 µM ATP (**A**), theoretical distributions at 3 µM ATP with n=2 (**B**), and theoretical distributions at 3 µM ATP with n=3 (**C**). Experimental distributions at 5 µM ATP (**D**), theoretical distributions at 5 µM ATP with n=2 (**E**), and theoretical distributions at 5 µM ATP with n=3 (**F**). The *X*-axis represents the number of ^18^O labeled oxygens (0, 1, 2, 3) in the released Pi. The initial distribution of the ^18^O label in the γ-phosphoryl group of ATP in these experiments was measured as follows—3 ^18^O: 57%; 2 ^18^O: 17%; 1 ^18^O: 2%; 0 ^18^O: 24%.

**Figure 10:**
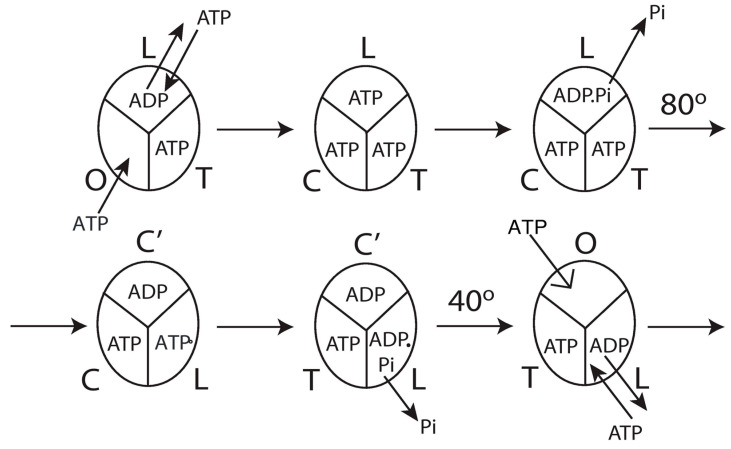
Model for steady-state multisite hydrolysis of ATP by F_1_-ATPase from the analysis of experimental oxygen exchange data by the stochastic kinetic theory in Section 3 and mechanistic interpretation based on Nath’s torsional mechanism of ATP synthesis/hydrolysis and the unified theory [18,30,31,32,33,57,62,63,64,81]. The three β-catalytic sites of the F_1_-ATPase enzyme are depicted; the system is being viewed from the F_1_-side. T represents the catalytic site of highest affinity for MgATP (site 1); L represents the catalytic site of intermediate affinity (site 2); O represents the site of lowest affinity (site 3). C′ stands for the conformation adopted by a closed catalytic site (which could even be half-closed, C) relative to the open (O) site. The trisite model depicts steady-state V_max_ hydrolysis with all three catalytic sites filled by MgATP or MgADP before rotation of the γ-subunit occurs. The open arrow in panel 6 shows a new MgATP molecule binding to site 3 (0°), following which the cycle repeats. See the text in Section 5.6 for details.

**Figure 11:**
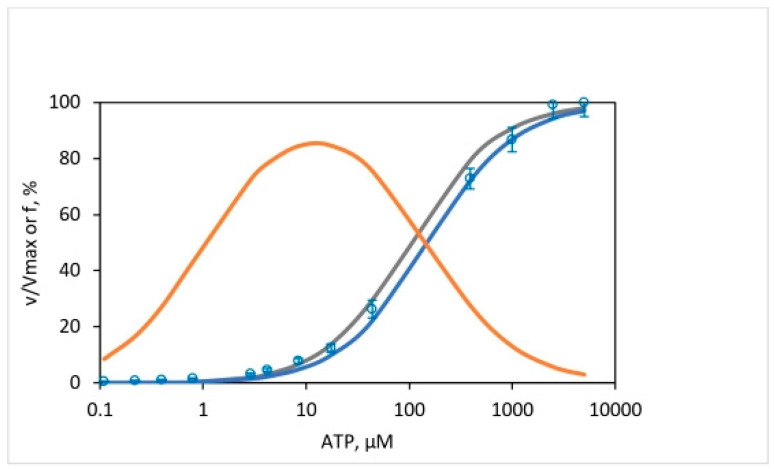
Fraction, f of total ATP hydrolysis activity by F_1_-ATPase (plotted as %) as a function of medium [ATP] concentration (from 0.11 to 5000 μM) contributed by the activity of (111) enzyme species, i.e., by species with all three catalytic sites occupied by Mg-nucleotide. Open blue circles (○) represent the experimental rates, v, normalized by our determined value of V_max_ for the mitochondrial ATPase of 640 ± 30 s^−1^ (*n* = 3). The gray curve shows the results of calculations using the K_d_ values of Weber, Senior, and coworkers [97] for sites 1 and 2 determined by their fluorescence binding technique—for the EF_1_, not the MF_1_–and our determined value of K_m_ for MF_1_ of 99 ± 15 µM, along with the good approximation that K_d3_ ≅ K_m_. The bold blue curve shows the results of calculations using the experimental values of K_d1_, K_d2_, and K_d3_ determined by Cross, Nalin, and coworkers on the MF_1_ [110,111]. The bold orange curve shows the results for a bisite model with (110) occupancies of catalytic sites.

The correspondence between the observed and theoretical [^18^O]Pi distributions (based on the stochastic kinetic theory in Section 3) shown in Figure 7 is fairly good but is not perfect. On close inspection, we noticed that the experimental [^18^O]P_3_ distribution relaxes at a much faster initial rate than that predicted theoretically (Figure 7A). Furthermore, the data clearly reveal that the [^18^O]P_2_ (Figure 7B) and [^18^O]P_1_ (Figure 7C) experimental distributions rise much faster at short times (0–~33 ms) than that predicted by theory. These trends are very regular, as seen from the experimental data plotted in Figure 7. Hence, although the long-time behavior (~33–266 ms) is well predicted, the rate of the short-time decay (for [^18^O]P_3_) or rise (for [^18^O]P_2_ and [^18^O]P_1_) is underestimated by the model. Hence we attempted prediction of the oxygen exchange based on the stochastic kinetic theory developed in Section 3 for *three* sites of oxygen exchange at short times (0–33 ms) corresponding to high substrate ATP concentrations (4.2–5000 μM) and *two* sites of oxygen exchange at long times (33–266 ms), i.e., at low substrate ATP concentrations (0.11–4.2 μM), at the experimentally-determined essentially constant value of k for oxygen exchange of 10.5 s^−1^ (Figure 4). The results are plotted in Figure 8 and in further experiments shown in Figure 9 that explore the region of intermediate [ATP] concentration. It should be clearly understood that the measured steady-state hydrolysis activity of F_1_-ATPase at every ATP concentration (µM to mM) arises entirely from the activity of the (111) species in a **trisite mode of catalysis** in which *rotation occurs with all three catalytic sites occupied by bound Mg-nucleotide* (Figure 10 and Figure 11).

The above results clearly show that all the four experimental ^18^O Pi distributions, i.e., [^18^O]P_3_ (Figure 8A), [^18^O]P_2_ (Figure 8B), [^18^O]P_1_ (Figure 8C), and [^18^O]P_0_ (Figure 8D) are perfectly predicted by the stochastic kinetic theory in Section 3 at both short times as well as long times, i.e., throughout the 50,000-fold range of medium ATP concentrations, without employing adjustable parameters. The finding of a constant value of the apparent rate constant of oxygen exchange determined from the measured overall extent of ^18^O/P (Figure 2 and Figure 4) and of a time of exchange that is twice the value determined from the reciprocal of the steady state hydrolysis rate at each medium ATP concentration at long times, and thrice that value at short times (Figure 8), offers novel mechanistic insights into catalysis by F_1_-ATPase. These, along with the biological implications arising, are discussed in Section 5. These findings also help in interpreting available high-resolution X-ray structures of F_1_-ATPase.

## 5. Discussion

### 5.1. Limits of Oxygen Exchange during Catalysis by F_1_-ATPase 

An upper limit of the ^18^O/P ratio of 3.98 was determined at low ATP concentrations (0.035–~0.1 μM), and a lower limit for oxygen exchange based on the ^18^O/P parameter of 1.01 was measured at high, saturating medium ATP concentrations (1–5 mM). 

### 5.2. Time-Resolved Analysis of Oxygen Exchange

The principal equations from our theoretical analysis in Section 3, Equations (6) and (13)–(16) enable a time-resolved analysis of oxygen exchange. Since the product of k and t, i.e., on the right-hand side of Equation (6), is readily evaluated from the ^18^O/P oxygen exchange data during ATP hydrolysis by F_1_-ATPase, no fitted parameters are required for the quantification and interpretation of the kinetic information. Equations (13)–(16) implement a Poisson-type model for the distribution of various [^18^O]Pi isotopomer species during ATP hydrolysis, and since these equations are also in terms of kt, no fitted parameters are necessary to predict these distributions.

### 5.3. Mechanistic Implications for Energy Coupling 

The stochastic kinetic model of the oxygen exchange process (Equations (6) and (13)–(16)) makes no specific assumptions about the mechanism and is therefore independent of the mechanism. These equations characterize the exchange in terms of the product of the apparent rate constant of exchange, k, and the lifetime of the exchanging species, t. Thus, a change in the ^18^O of Pi could arise either from an alteration in the rate constant of exchange or from an increase in the time available for exchange. The latter situation would arise due to the differing length of time spent by the Pi/ATP in the β-catalytic site due to a faster/slower turnover of the hydrolytic reaction. For instance, a longer time spent while bound ATP is waiting for rotation of the γ-subunit/conformational change in F_1_ to take place before activation or before the cleavage chemical step occurs at a catalytic site to form Pi, or a longer time spent while the Pi formed after hydrolysis is waiting for its release from the site etc., will show up as an increased extent of oxygen exchange at a lower ATP concentration.

Different models of energy coupling have been constructed based on their fundamental conception of oxygen exchange, where the parameter chiefly modulates the exchange with changes in medium/substrate concentration. The binding change mechanism of ATP synthesis does not include a timescale for the exchange per se; it focuses on the alteration of the value of the rate constant governing the oxygen exchange, k, of nucleotide bound to a catalytic site by the medium substrate concentration. The mechanism proposes long-range transmission of the binding energy of ATP from one β-catalytic site via the ring of β and α subunits to another β-catalytic site (site-site cooperativity) in F_1_-ATPase and modification in the value of the rate constant of the exchange by such signal and energy transmission [58,60]. In their words, emphasis is placed on “how much rate constants at one catalytic site may be changed by ATP binding at another” [58]. The torsional mechanism of energy transduction and ATP synthesis and the unified theory of ATP synthesis/hydrolysis [31,57] emphasizes the asymmetric interactions of the β-catalytic sites with the single copy γ- and ε-subunits during catalysis [33,62,63,64]. It considers the time available for exchange, t, by the bound ATP/Pi at each catalytic site as the appropriate timescale and postulates that this parameter quantitatively determines the extent of the oxygen exchange observed in the experiments when multiplied by an essentially constant apparent rate constant for the exchange process, k. According to this theory, changes in the medium substrate concentration alters t at each of the multiple sites where the oxygen exchange occurs, which is then responsible for the modulation of the extent and rate of oxygen exchange in accordance with Equations (6) and (13)–(16). 

The results shown in Figure 4 and Figure 8 demonstrate that the measured changes in the extent of ^18^O as a function of medium ATP (from sub-micromolar to millimolar concentration) incorporated into Pi by an intermediate Pi–HOH oxygen exchange accompanying ATP hydrolysis arise entirely from differences in the time available for exchange rather than from any alteration in the value of the rate constant of ^18^O exchange over the entire 50,000-fold concentration range of substrate. 

To sum up, the results prove that consideration of the time available for ^18^O exchange by bound nucleotide/Pi in a catalytic site at any substrate ATP concentration is sufficient to account quantitatively for the observed extent of oxygen exchange by F_1_-ATPase, without the need for postulating additional allosteric interactions/site-site cooperativity between catalytic sites. It should be noted that it is imperative fundamentally to account for changes in the timescale t arising from substrate concentration changes imposed by the experimentalist. Once this effect is exactly accounted for (Figure 4), there is no leeway for postulating other effects such as site-site cooperativity/allostery.

### 5.4. Mechanism of Oxygen Exchange

Another fundamental limitation of the model of oxygen exchange proposed by the binding change mechanism was that it involved only a single site or route of entry of water oxygen. Previous research did not report or quantitatively tabulate the absolute rates and rate constants of the exchange of ^18^O between P_i_/ATP and HOH in a model-independent way. Thus, any observed extent of exchange was rationalized by assuming reversible ATP synthesis/hydrolysis at a single catalytic site with an equilibrium constant of $K_{eq}≅1$, and postulating the number of reversals *that must have occurred at the catalytic site* to account for the measured extent of exchange, assuming a ¾ probability of loss/gain of the ^18^O label with each reversal. Thus, at low substrate ATP concentration, more than 400 reversals of the reaction had been postulated to explain the observed extent of oxygen exchange [58]. This view of spontaneous and reversible catalysis has been challenged by several workers [33,34,82]. Moreover, no independent verification of the number of reversals occurring during catalysis has been provided by the proponents of the binding change mechanism. Furthermore, the original postulate of the binding change mechanism of $K_{eq}≅1$ was never supported by the experiment. It was even admitted by the workers that “Measurement of the equilibrium constant for the reversible hydrolysis of bound ATP at high ATP concentrations would be useful but does not appear to be readily accessible experimentally” [58]. 

A major finding of this work (Section 4.4) shows that there are *multiple* sites of oxygen exchange during the catalysis of ATP synthesis/hydrolysis, as predicted by the torsional mechanism [32]. We could not match the observed [^18^O]Pi distributions with a single site/route of entry of ^18^O water (Figure 6). Hence, our results cannot be explained by the binding change mechanism, and a new mechanism of oxygen exchange is required. We have advanced a mechanism according to which the exchanges do not involve the spontaneous reversal of the formation of ATP from ADP and Pi at a catalytic site but occur instead because the catalytic site of the enzyme lacks absolute spatial selectivity for the oxygen ligands of the phosphorus intermediate that it accepts and binds as substrate [57,81]. Given the above, it is clear that ligands can readily permute at an intermediate state and that the oxygen ligands can *interchange* their positions about the phosphorus center. We propose here that such **ligand permutation** is the fundamental cause for the occurrence of the intermediate Pi–HOH (and the intermediate ATP–HOH oxygen exchange during ATP synthesis by the F_O_F_1_-ATP synthase [57]), as opposed to multiple spontaneous reversals of ATP synthesis/hydrolysis at a catalytic site [58]. Further, we envisage that such ligand permutation in space about the bound Pi/γ-phosphoryl of ATP occurs under conditions of tight electrostatic interactions with catalytic site groups so that the electrostatic free energy released during the process of ATP hydrolysis can be readily transduced into the torsional energy stored in the γ-subunit of ATP synthase, as per the postulates of the torsional mechanism of energy transduction and ATP synthesis/hydrolysis and the unified theory of ATP synthesis/hydrolysis [30,31,33, 62−64,81]. Mg^2+^ plays a critical role in this process, as proposed previously [63,64,83,84].

It is clear from the above discussion that the free energy required for the ATP synthesis process is obtained from the torsional energy stored in the γ-subunit, which is ultimately derived from the energy of the anion and proton electrochemical gradients (Δμ̃_A_^−^ + Δμ̃_H_^+^) [85,86,87,88,89]. A unified treatment of coupling [90] and uncoupling [91] during the process of oxidative phosphorylation that employs the same mathematical equations has also been developed.

### 5.5. Number of Sites of Oxygen Exchange and Their Biological Implications

The results presented in the latter part of Section 4.4 and plotted in Figure 8 pose an interesting mechanistic conundrum. How can we have three catalytic sites participating in oxygen exchange *at short times* that, however, reduce to two sites mediating intermediate Pi–HOH exchange *at long times*? In other words, at long times, one catalytic site drops out from the exchange from the maximum value of the number of catalytic sites, n=3, mediating oxygen exchange at short times. This fact further imposes severe constraints on possible molecular mechanisms of ATP hydrolysis by F_1_-ATPase.

Every model of trisite hydrolysis, when looked at in a gross way, postulates for the enzyme as a whole that site 3 (O) changes to site 1 (T), site 1 (T) changes to site 2 (L), and site 2 (L) changes conformation to site 3 (O). Following the conformational change to site 3 (O), the bound nucleotide would be released and, therefore, can no longer participate in the exchange. If two catalytic sites have to contribute equally to exchange at long times by effectively doubling the time of exchange (Figure 8), then these have to be sites 1 and 3 that have ATP bound to them, and they need to continue to exchange oxygen during the O → T and T → L transitions of the respective sites to explain the observed [^18^O]Pi distributions at long times. If site 2 had contributed at long times, we would obtain a non-integer value for the exchange; moreover, if site 2 contains ADP as expected, then it should not mediate any exchange in the first place. By another element of logic, the site that could contribute to exchange at short times (and subsequently not make a contribution) has to be the site (site 2) that engages in the L → O change.

The above logic, though compelling, still does not solve the conundrum. The L-site initiates the 80° sub-step of γ rotation by activation of the system by ATP binding, or rather, by ADP–ATP exchange in site 2 by **ligand displacement** as proposed by us [31] (since the site contained bound ADP initially). Now, site 2 contains bound ATP, but if the bound ATP does not exit after the L → O conformational change and continues to exchange oxygen, then n=3 always, which is not consistent with the experiment. If the ATP unbinds and exits the site after its L → O transition, and a new ATP does not bind, then n=3 is not realized even at short times. If a new molecule of ATP binds in O, then the L-site that had mediated an ADP–ATP exchange by ligand substitution shall only mediate an ATP–HOH exchange but not show up as an intermediate Pi–HOH exchange. Hence, again, we can never get three sites engaged in a Pi–HOH exchange. Again, the conundrum is not solved fully.

Thus, to have three sites mediating an intermediate Pi–HOH exchange at short times, we need to additionally *hydrolyze* the bound ATP (that had exchanged with bound ADP in L, i.e., site 2 by the phenomenon of ligand displacement) to ADP.Pi, and subsequently, Pi needs to unbind and be released from L. This explains the short-time exchange data (Figure 8). The rate of this activation and ligand displacement phenomenon is itself inversely proportional to medium ATP concentration, as one would expect. Thereafter, L shall only contain bound ADP, which does not mediate oxygen exchange with water, and therefore, the number of catalytic sites mediating exchange, n=2 at long times. Subsequently, the bound ADP is released after an L → O transition of the catalytic site, and a new ATP binds to O, which thereafter adopts a closed conformation (relative to O) and participates in oxygen exchange as the site changes to T, and continues to mediate exchange as T changes to L. Following the site’s T → L transition, ATP hydrolyzes to ADP.Pi in L, and after that, Pi continues the exchange process, which ceases only upon the release of Pi from L. This explains both the short and long-time exchange data, predicts the experimental [^18^O]Pi isotopomer distributions with 3, 2, 1, and 0 ^18^O atoms over the entire range of medium ATP concentration, and resolves the conundrum posed at the beginning of this section.

The above explanation also serves to rationalize our puzzling biochemical observations on MF_1_ that when normal nonradioactive ATP is pre-loaded in the highest affinity site 1 (T) under sub-stoichiometric ATP:F_1_ conditions and 5–20 μM radioactive [γ-^32^P]ATP is used in a cold chase experiment, 92–94% of the promoter [γ-^32^P]ATP is hydrolyzed in site 2 (L) within 10 s of incubation time, as detected by counting of ^32^Pi. This also leads to the key insight that ATP hydrolysis and Pi release (and not ATP binding as in previous theories of free energy transduction [59,60]) in site 2 is also required to explain the chase promotion experiments; otherwise, no ^32^Pi counts should have been registered in the above experiment [92].

Figure 9 shows the results of our experiments on hydrolysis by mitochondrial F_1_ on the [^18^O]Pi distributions that further explore the range of intermediate (~μM) ATP concentrations. The results show that between 5 µM and 3 µM [ATP], the number of catalytic sites mediating oxygen exchange at the same time switches from three (Figure 9D–F) to two (Figure 9A–C), respectively. Alternatively, more precisely, the intermediate Pi–HOH oxygen exchange occurring in site 2 decays to zero and ceases after ~33 ms, and hence, that catalytic site drops off from the observed exchange at longer times (~33–266 ms). Hence, there is a transition from n=3 to n=2 between 3 and 5 µM [ATP], and only then can the detailed [^18^O]Pi distributions shown in Figure 8 and Figure 9 be predicted exactly. 

Single-molecule recordings on F_1_-ATPase do not measure the extensive oxygen exchange that occurs at multiple catalytic sites at the same time before, during, and after ATP cleavage. However, the above results are not in conflict with results from the single-molecule observations. An exchange rate of 10.5 per s (Figure 4) implies that, on average, an exchange occurs once every 100 ms. At the transition concentration between 3 and 5 µM ATP discussed above, the results of Figure 8 and Figure 9 show that the oxygen exchange process in site 2 lasts ~33 ms. For this entire duration of ~33 ms, the bound MgATP molecules in sites 1 and 3 also participate in the exchange process, and the extent of exchange that occurs is approximately equivalent to that by a single catalytic site involved in the exchange process for ~100 ms. Approximately because further exchange occurs at the intermediate stage of the reaction and afterward when MgADP.Pi remains bound, and the Pi is waiting to be unbound and released into the medium. It should be clearly understood that the ATP cleavage reaction takes place at one catalytic site at a time; hydrolysis does not occur simultaneously at two or three sites, although oxygen exchange does. Furthermore, the ATP hydrolysis (bond cleavage) event can occur at a much higher rate, with a $t_{1/2}$ of ~1 ms during the catalytic dwell, as revealed by molecular dynamics simulations [25] and single-molecule experiments [11,14,93,94,95,96], since much of the oxygen exchange occurs when MgATP is bound at multiple catalytic sites for a characteristic time as a function of medium ATP concentration, and becomes longer as ATP concentration decreases. 

### 5.6. Model for Steady-State Multisite ATP Hydrolysis by F_1_-ATPase 

The detailed molecular mechanism of V_max_ ATP hydrolysis by F_1_-ATPase ([31], pp. 1795–1797 and pp. 1802–1805) can now be refined to make it consistent with the results obtained in Section 4.1, Section 4.2, Section 4.3 and Section 4.4, and the mechanistic implications of biological energy transduction arrived at in Section 5.1, Section 5.2, Section 5.3, Section 5.4 and Section 5.5. Such a model is illustrated in Figure 10.

The molecular mechanism of ATP hydrolysis by F_1_-ATPase (Figure 10) incorporates a key result arising from this work that it is not sufficient to exchange bound ADP in the catalytic site 2 (L) with medium ATP in order to *activate* the enzyme and cause an 80° primary rotation of the central γ-subunit (in a clockwise sense when viewed from the F_1_ side) in F_1_ (**bisite activation**) We additionally need to *hydrolyze* the bound ATP in site 2 (L) (that had exchanged with ADP in the catalytic site) to ADP.Pi and subsequently the Pi need to move away and be released from L, as formulated by a general theory of biological energy transfer [30]. Otherwise, the ^18^O labels would not have been detected by mass spectrometry in the Pi released into the medium, and we would not be able to explain the distribution of [^18^O]Pi species (Figure 8). Thus, this L-site is the third catalytic site at which exchange occurs and, therefore, accounts for the observation of n=3 at short times. The exchange of bound MgATP for MgADP in site 2 releases an excess binding energy of ~9 kJ/mol in the F_1_-ATPase, i.e., the difference between the binding energy of MgATP in L (~36 kJ/mol) and the binding energy of MgADP in L (~27 kJ/mol). This ~9 kJ/mol energy released weakens to approximately zero the binding of bound Pi formed upon the ATP hydrolysis event in site 2 (i.e., cleavage of the P_β_–O–P_γ_ terminal phosphoanhydride bond of the ATP originally at a bond distance of 0.3 nm [30]). The effect of ejecting ADP with a certain velocity helps break the ~9 kJ/mol γ–β_TP_ interactions, i.e., between γ and site 2. Now, the γ-subunit is free to rotate, and the Pi is free to move away from bound MgADP. As the Pi moves step-wise from 0.3 to 0.4 to 0.6 nm [30], it releases a Coulombic repulsion energy of 9 + 9 = 18 kJ/mol. Another ~18 kJ/mol is made available as the Pi is fired out from 0.6 nm to ∞ and released into the solution. This ~36 kJ/mol electrostatic potential energy rotates the top of the γ-subunit by 80° relative to the stationary β–subunits with an average torque measuring ~40 pN-nm generated at the β–γ interface at a radial distance of approximately 1 nm from the central axis of the α_3_β_3_ hexamer (Figure 10).

After initiation of the 80° rotation of the top of γ, site 2 (L) changes to a closed conformation C′. Upon the above 80° rotation, the top of γ interacts with the β-catalytic site 1 (T or β_DP-like_ in [31]) and alters its conformation to loose (i.e., site 2, L or β_TP_ in [31]). In other words, the rotation of γ_top_ causes a T → L transition of the β-catalytic site, due to which a ~9 kJ/mol destabilization (reduction in binding energy of intermediate bound in the site) occurs. Concomitantly, ATP hydrolyzes to ADP.Pi upon the T → L transition of the catalytic site. Pi, which is bound to L with ~9 kJ/mol binding energy, is now bound in L with ~zero binding energy and is, therefore, free to move away. The 0.3 → 0.4 → 0.6 nm movement of Pi away from bound ADP releases ~18 kJ/mol, which is transmitted from site 2 to site 3 (O or β_E_) along the ε-helix and helps break the ε–β_E_ interaction (e.g., between ε–Ser-108 and β_E_–Glu-381 in the DELSEED loop) [33] along with the ~27 kJ/mol binding energy of MgATP in site 3 (O or β_E_), as already described [31]. The open site O or β_E_ closes (to β_C_) due to the interactions mentioned above and relieves any steric hindrance that the open site offered to further rotation of the γ-subunit (beyond 80°). The torsional strain in the γ-subunit also helps break the ε–Ser-108–β_E_ and the ε–Met-138–β_TP_ interactions and rotate the ε-subunit (both the N-terminal β-sandwich domain and the C-terminal helical domain) and the bottom of the γ-subunit by 80°—clockwise as seen from the F_1_ side—and the two-coiled coil α-helices of γ unwind, relieving the torsional strain in the γ-subunit. This is accompanied by the concomitant transition O → T of the catalytic site. In the meantime, the release of Pi from the new L-site (i.e., the one that has become site 2 after its T → L transition owing to the 80° rotation of the top of γ already described above) with a velocity upon its movement from 0.6 nm away from ADP to infinity provides the energy for the remaining 40° rotation of the γ- and ε-subunits (Figure 10). This event of Pi release from the new β_TP_ (after the ~80° rotation, or at an angular position of ~200° after the ATP molecule binds at 0°) into the medium is the one that finally leads to the cessation of the intermediate Pi–HOH exchange. It also accounts for n=2 observed at long times arising from oxygen exchange in site C and during C → T, and concomitantly in T and during T → L until Pi is released from L. Upon this 40° rotation, the interaction of ε with C′ changes its conformation to an O-site (open, site 3, the site with the lowest affinity for Mg-nucleotide) from which bound MgADP is released. During steady-state V_max_ hydrolysis, the order of conformations that a *single* catalytic site of F_1_-ATPase passes through is O → T, T → L, L → C′, and C′ → O. Looking at the enzyme *as a whole*, the order of the conformational changes of the catalytic sites during multi-site hydrolysis by F_1_-ATPase is L → C′, followed by T → L, followed by O → T, and finally, C′ → O, in accordance with our previous predictions, and also shown to be the microscopic reversal of the ATP synthesis cycle ([31], pp. 1801, 1804). The cycle (Figure 10) then repeats.

The catalytic cycle for steady-state V_max_ hydrolysis depicted in Figure 10 is in accord with biochemical crosslinking studies [16,17]. These studies inferred from the data that rotation of the γ- and ε-subunits in F_1_-ATPase is not linked to the unisite hydrolysis of ATP at the highest affinity catalytic site 1 (T) but to ATP binding and/or ATP hydrolysis and product release at the second or third catalytic site on the enzyme (i.e., site 2 or site 3) [16]. The studies also showed that the effect of covalently crosslinking β–Cys-381 to γ–Cys-87, i.e., forming the β–γ crosslink, increased the rate of unisite catalysis to that obtained by the cold chase of ATP of the non-crosslinked enzyme [16]. Since β–γ in the biochemical crosslinking studies corresponds to β_TP_ in the X-ray structure of the enzyme in the Mg-inhibited state [1], we infer that β_TP_ (site 2 or L) is the catalytic site to which ATP binds (and in which it subsequently hydrolyzes) in the native non-crosslinked enzyme. These events are responsible for rotating γ by 80°, changing the conformation of site 1 to site 2, and causing hydrolysis of the bound ATP in the (new) site 2, as shown in Figure 10.

The molecular mechanism shown in Figure 10 also satisfies the fact that V_max_ ATP hydrolysis follows trisite catalysis [32,33,34,97], a fact that is considered experimentally proven in our study. This by itself takes it beyond the binding change mechanism, which was necessarily a bisite model [60,61]. However, over the past two decades [29,34] until the present day [7], ATP binding to site 3 (O) has been repeatedly postulated to cause rotation in F_1_-ATPase. It has been pointed out previously that the O-site (site 3) is open and distorted, and the binding energy of MgATP is only ~27 kJ/mol [31,32,97], which is grossly insufficient energetically to change the conformation of the catalytic site from O to closed (C) and also cause a primary rotation of the γ- and ε-subunits by 80° (see p. 1809 of [31]). It ought to also be stressed that the detailed mechanism of steady-state multisite ATP hydrolysis by F_1_-ATPase presented here (Figure 10) is the *microscopic reverse* of the molecular mechanism of steady-state ATP synthesis by F_O_F_1_-ATP synthase formulated by us in previous publications [31,33,57]. The fundamental constraint of microscopic reversibility has not been shown to be satisfied by other mechanisms. For these compelling reasons, we consider the mechanism shown in Figure 10 to be superior to extant mechanisms in the field.

### 5.7. Order of Product Release Steps during ATP Hydrolysis and Interpretation in Terms of High-Resolution MF_1_ X-ray Structures

The order of release of products during ATP hydrolysis by F_1_-ATPase of Pi followed by ADP in Section 5.6 and Figure 10 is consistent with the finding from the MF_1_ X-ray structures that ADP is the last of the products of ATP hydrolysis to be released [98] (PDBID: 4ASU). Mechanisms that propose the reverse order, with Pi release following ADP release from a catalytic site, are faced with the difficulty of retaining Pi for an additional 120° after hydrolysis when the catalytic site’s affinity for Pi is already very low. How does the Pi sense the timing of its release? No convincing proposal that overcomes the above difficulties has been put forth. Above all, such a proposal would not be in agreement with the [^18^O]Pi isotopomer distributions of Figure 8 at long times. Since Pi release necessarily follows ATP bond cleavage in our scheme of coupling (Figure 10), these serious difficulties are avoided by our mechanism (Section 5.6).

Our proposed mechanism and order of product release during ATP hydrolysis is also consistent with our other biochemical findings on mitochondrial F_1_. For instance, when [γ-^32^P]ATP is used as a substrate for MF_1_-ATPase, we found that the ratio of bound ^32^Pi to that of bound ^32^Pi and bound [γ-^32^P]ATP remained constant at 0.333. This distribution of product to substrate was maintained at different incubation times of the reaction mixtures between 1 and 15 min [92]. The same invariant value of the ratio was found for various 1:1 concentrations of MF_1_ to substrate in the range tested between 0.5 and 1 μM. This observed distribution between the bound product and bound substrate is very difficult to explain using other models.

Finally, the above order of product release is in accord with that found by single-molecule studies on F_1_-ATPase [11,15] and in other ATP-hydrolyzing systems such as purified myosin II [99] and unconventional myosin V [23] motors, and also with kinetic studies on muscle contraction [100,101,102]. 

### 5.8. Identity of Site 2 in the MF_1_ X-ray Structures

Suppose we were to reverse the identity of sites 1 and 2 in Figure 10 for ATP hydrolysis by F_1_-ATPase. Let the catalytic site assigned as site 2 or L (β_TP_) in panel 1 of Figure 10 be identified as site 1, and the site designated as site 1 or T (β_DP_ or β_DP-like_) in Figure 10 be identified as site 2. Then, in the next conformational change after the ~80–90° clockwise rotation of γ, O, i.e., site 3 or β_E_ changes to β_DP-like_ (now assigned as L or site 2), β_DP-like_ changes to β_TP_ (now ascribed to be T or site 1), and β_TP_ changes to β_E_. That is, in the reversed assignment, O → L, L → T, and T → O. However, this is neither in accordance with our cold chase experiments nor with trisite hydrolysis in which O should change to T, T to L, and L to O. It implies that the reversed identity assumed at the beginning of this paragraph is incorrect. Moreover, the reversed identity of the sites is unable to reproduce the configuration of the enzyme in Figure 3 of the original paper depicting the Leslie–Walker structure in the MgADP-inhibited state [1]. Hence, β_TP_ in the Leslie–Walker structure [1] is site 2, as proposed originally by the proponents [1,103] (PDBID: 1BMF, 1W0J, 1W0K), and also concluded by us from analysis of the architecture of the catalytic sites [32,62,63,64]. 

Our conclusion that “unisite” hydrolysis occurs in site 2 (β_TP_) is also in agreement with the finding of an important recent cryo-EM structural study carried out under various reaction conditions [7] (PDBID: 7XKH, 7XKO, 7XKP, 7XKQ, 7XKR).

The important phenomenon of ligand displacement also explains why the β_TP_ site does not contain bound MgADP in the original high-resolution MF_1_ X-ray structure. Since, according to our mechanism, the ATP (or its analog) displaces bound ADP from site 2 (Section 5.6), β_TP_ should contain bound AMP-PNP, as found [1]. Our mechanism also helps understand why no MF_1_ structure with ATP or ATP analog bound to the β_DP_ (or β_DP-like_) subunit has been solved and why most MF_1_ structures capture an ~80–90° rotated state of the enzyme (PDBID: 1BMF, 1H8E) [1,2] (the so-called Mg-inhibited state [1] or the catalytic dwell [11,14,15]) as opposed to a 0° resting state (panel 1 in Figure 10) (the so-called ATP-waiting dwell/state [11,14]). The ADP–ATP exchange and ATP hydrolysis followed by Pi release due to cold chase in site 2 will immediately rotate γ by ~80–90° and transform the β_DP_ (or β_DP-like_) site to β_TP_, where the bound ATP gets hydrolyzed, as explained in consummate detail in Section 5.6, which will be captured by the crystal structure.

### 5.9. Angular Position of ATP Binding, Bond Cleavage, Pi Release, and ADP Release during ATP Hydrolysis by F_1_-ATPase

Looking at a single catalytic site, MgATP binds to O (site 3 or β_E_) at 0°, which becomes T (site 1 or β_DP-like_) [31] after the ε–subunit moves away during the 0 → 120° rotation of γ–ε. The bound MgATP is hydrolyzed at 200**°** due to its conformation change from β_DP-like_ to β_TP_ (site 2), i.e., owing to a T → L transition of the site. Pi is then released from L at 200°, leading to a 40° rotary sub-step (Figure 10, Section 5.6). The ADP unbinds from L at 240° and is fired out because the ADP is *displaced* by medium ATP, which now binds in L (**ligand substitution**). However, the L-site is meant for ADP.Pi, and therefore, the ATP immediately hydrolyzes in site 2 (L), following which Pi is released (“unisite” catalysis in site 2), which gives energy for the 80° rotation sub-step. The L-site now changes to a closed site. The interaction of the C-terminal of the rotated ε–subunit with the closed site induces a conformational change of the catalytic site to its open (O) conformation from which the bound MgADP is unbound and released. A new MgATP binds to O, and the cycle repeats.

To summarize, the elementary chemical processes and the angular position at which they occur during ATP hydrolysis by F_1_-ATPase that are consistent with our oxygen exchange analysis are as follows: ATP binding—**0°**, ATP bond cleavage—**200°**, Pi release—**200°**, and ADP release—**240°**. 

The above correlation of the timing of elementary chemical processes in the F_1_-ATPase with rotary angle is in agreement with the latest biochemical study (2023) in which Nishizaka and coworkers generated a hybrid α_3_β_3_γ subcomplex in F_1_ from thermophilic *Bacillus* PS3 that consisted of one mutant β and two wild type βs [15]. The enzyme carried a β(E190D/F414E/F420E) mutation, which caused extremely slow rates of both ATP cleavage and ATP binding that enabled unequivocal determination of the angular position of the ATP cleavage reaction (200°) after ATP binding at 0° [15]. However, these studies do not explain how and why such a coupling scheme is operative, and of course, they cannot give the detailed molecular mechanism and dynamics of the process as done here.

### 5.10. Consistency of the Proposed Mechanism with the Results of Catalytic Site Nucleotide Occupancies of Senior and Colleagues Using Tryptophan Fluorescence Quenching [97,104,105,106] and Relationship with Other Models of ATP Hydrolysis [2,7,14,15,31,38,82,93,96,107,108]

Senior, Weber, and colleagues engineered a mutant *Escherichia coli* F_1_ in which the tyrosine residue of β-subunits, Tyr-331, was replaced by a tryptophan residue [104]. They showed that the tryptophan fluorescence was quenched proportionally as Mg-nucleotide bound to the β-catalytic sites and that the signal in the βY331W F_1_ could be reliably used to monitor occupancy of the sites with substrate MgATP. The present author was the first scientist to recognize this experimental breakthrough and laud it in the following words ([33], p. 73), “Recently, in a significant development, Senior and colleagues designed an optical probe by inserting a tryptophan residue to directly monitor, for the first time, the occupancy of the catalytic sites by nucleotides. Their tryptophan fluorescence experiments in the hydrolysis mode established definitively that steady-state V_max_ activity is attained by F_1_-ATPase only when all three catalytic sites are occupied by bound nucleotide, i.e., an enzyme species with all three β subunits occupied is the only catalytically competent species” [97,104,105,106]. 

The use of a second Trp mutant at β–Phe-148 showed definitively that the fluorescence signals were different with bound MgADP as compared to bound MgATP in catalytic sites. The workers found that, on average, two molecules of ADP and one molecule of ATP were bound to catalytic sites during steady-state hydrolysis by F_1_-ATPase [105]. Further, the experiments showed that P_i_ binding to catalytic sites cannot be spontaneous, as in models [60,109] of the binding change mechanism, which must therefore be incorrect (see also pp. 71–75 and Figures 2 and 3 in ref. [33]). Rather, there is a requirement for energy input from the ion gradients to enable Pi binding in the ATP synthesis mode [106] and a fundamental requirement for a mechanism of Pi activation in ATP synthesis [31,57].

Senior and coworkers believed that the exchange results that Boyer obtained [58] were only relevant at the lower ATP concentrations [34]. In their words, “the ^18^O exchange experiments are probably monitoring a special activity that occurs only at low nucleotide occupancy of catalytic sites, or in the absence of rotation, but is not reflective of physiological steady-state catalysis” [34]. However, this was only a qualitative surmise, and no results were presented in support of their view. The results of our work show that the ^18^O exchange methodology is indeed capable of providing valuable information on mechanisms over the entire concentration range from low to high [ATP] (Figure 2, Figure 3 and Figure 4 and Figure 8) if a quantitative model of exchange had been developed as done in Section 3 and Reference [57], and the results interpreted correctly using such a stochastic kinetic model. This can be subjected to quantitative testing, as explained below. 

In the oxygen exchange experiments presented here over the 50,000-fold range of ATP, we can calculate the extent of nucleotide occupancy at catalytic sites using Senior’s data [97] and determine how that impacts the results and conclusions of this work. Figure 11 shows representative results of this exercise. The open circles show the experimentally determined enzyme activity of MF_1_ for [ATP] from 0.11 to 5000 μM. The data are normalized by our value for V_max_ determined for the mitochondrial ATPase of 640 ± 30 s^−1^. The calculated curves show the fraction of catalytic sites with **trisite filling**, i.e., with all three sites occupied by Mg-nucleotide during hydrolysis. These calculations are given for two sets of values of the dissociation constants (K_d_) of sites 1, 2, and 3. The bold gray curve uses the K_d_ values tabulated by Senior and coworkers [97] obtained by their true “equilibrium” (i.e., when Mg-nucleotide binding to catalytic sites has reached a steady state) fluorescence binding technique for sites 1 and 2 of 0.02 and 1.4 µM, respectively—which are for the EF_1_, not the MF_1_—and our determined value of K_m_ for MF_1_ of 99 ± 15 µM, with the very good approximation that K_d3_ ≅ K_m_. The bold blue curve shows calculation results with the values of K_d1_, K_d2_, and K_d3_ of 0.018, 1, and 150 µM, respectively, measured by Cross, Nalin, and coworkers on MF_1_ [110,111]. In general, the finding arises that the binding of MgATP to site 3 is weaker in MF_1_ compared to that in EF_1_, as shown by values of the dissociation constant in site 3, K_d3_ in the range of 100–150 µM and 23–30 µM for MF_1_ and EF_1_ respectively. Figure 11 shows conclusively that a trisite mechanism of ATP hydrolysis is the operative mode of catalysis over five decades of ATP concentration. Models with (110) bisite nucleotide occupancies do not fit the enzymological data of Figure 11 (bold blue circles vs. bold orange curve that uses the experimental K_d_ values measured for MF_1_ [110,111]), similar to their inability to predict the oxygen exchange data (for example, compare Figure 6 with Figure 8). The use of all possible bisite enzyme species (i.e., occupancies due to (110) + (101) + (011) species) gave similar results to that modeled by the bold orange curve in Figure 11. Results for the trisite vs. bisite models using experimental data on the *E. coli* F_1_-ATPase and K_d_ values for the three catalytic sites of EF_1_ measured by Weber and Senior [97] have already been published (see Figure 2 of ref. [32]).

The results shown in Figure 11 have major biological implications. First, they show that the results of the ^18^O exchange are not limited to the low [ATP] range as suggested previously [34] but also apply to the physiologically important limit of mM [ATP] and that a single mechanism operates throughout this range, as also found in a single-molecule study by the Kinosita group [11]. Second, it provides us with another yardstick to discriminate between bisite models, like Boyer’s binding change mechanism, and trisite models, such as Nath’s torsional mechanism of energy transduction and ATP synthesis/hydrolysis, and critically appraise models of ATP hydrolysis by F_1_-ATPase proposed on the basis of single-molecule experiments.

A large number of technologically sophisticated single-molecule studies on F_1_ have been carried out by eminent Japanese researchers over the past 25 years [14,15,93,94,95,96], witnessed first-hand by the author during his sabbatical year at Waseda University, Tokyo [92]. Various models of ATP hydrolysis by F_1_-ATPase have been proposed based on these studies. However, in what must be regarded as a travesty that also points to the inherently elusive nature of the conundrum of mechanochemical coupling, the models are effectively bisite, and *none of these models, based on single-molecule data, without exception, incorporates a true trisite mechanism*. Therefore, they contradict Senior’s experimental data [97,104,105,106] and our results shown in Figure 8 and Figure 11. 

As mentioned above, the models proposed for ATP hydrolysis by F_1_-ATPase based on single-molecule studies contradict experimental data based on direct, real-time monitoring of catalytic site nucleotide occupancies [97,104,105,106]. In these models, rotation takes place with two sites occupied by Mg-nucleotide; for example, in the model of References [93,95] that postulated for the α_3_β_3_γ subcomplex of TF_1_ from thermophilic *Bacillus* PS3, both the 80° and the 40° sub-steps of the rotation of the γ-subunit take place in bisite mode. In the model of Reference [96] for the complete human mitochondrial F_1_-ATPase, all three sub-steps for rotation of γ-ε from 0–65°, 65–90°, and 90–120° postulated to be driven by ATP binding, Pi release, and ATP bond cleavage, respectively [96], occur with two sites containing bound Mg-nucleotide. For bovine MF_1_, a bisite mode of catalysis is suggested for rotation of γ-ε for the substep from (at least) 10–20° to 80° and for the 80–120° substep [14]. Models that propose concerted ATP binding to a site and ADP release from a different site [14,95,96] as driving rotation cannot be trisite. These models are incorrect, given that the operative mode of catalysis during steady-state ATP hydrolysis by F_1_-ATPase is trisite. A frequently used definition of ATPase mechanism as trisite, because it alternates between having two and three catalytic sites filled with nucleotide at any time, is inadequate. Based on this imperfect criterion, even the binding change mechanism as modified by Cross is trisite [109]! For a mechanism to be truly trisite, catalysis must occur, and rotation must take place during steady-state V_max_ hydrolysis *only when all three catalytic sites are occupied by bound Mg-nucleotide* [97,104,105,106].

In our view, careful attention needs to be paid to theoretical developments that are based on fundamental physicochemical considerations. For example, it was shown that the electrostatic potential energy obtained upon Pi movement away from bound MgADP and its release from an enzyme catalytic site can be used to weaken a bond, surmount energy barriers in conformational changes, store internal energy in a local domain of the enzyme, or perform useful external mechanical work [30]. Such a fundamental theory can help solve the sturdy problems of molecular mechanisms that have caused major headaches to several researchers in the field. Another approach is to realize that the above anomalies arise primarily from the fact that while single-molecule studies monitor rotation, say of the central γ- or γ-ε subunit on which the optical probe is bound with unprecedented resolution, they do not directly monitor catalytic events in the β-subunits, and therefore they are blind to catalytic site nucleotide occupancies. Hence, a new high-resolution hybrid experimental technique may be required. Most importantly, all the available experimental information needs to be integrated into a single consistent model. This has been the attempt of the torsional mechanism and unified theory.

The converse is also true. Thus, studies that directly monitored nucleotide occupancies of β-subunits proposed models of ATP hydrolysis by F_1_-ATPase [97,104,105,106] contradict results from decades of single-molecule work [14,15,93,94,95,96]. The former studies correctly inferred that for a trisite mechanism to operate, ATP binding to site 3 with a K_d3_ value of ~100 µM is too small to provide the required quantum of energy to drive the 80° rotational sub-step. Hence, they proposed that ATP binding to site 3 followed by ATP hydrolysis in site 1 acting in sequence provide energy for the 80° sub-step of γ rotation [34,97]. The problem for these models is that single-molecule experiments on F_1_ have unequivocally shown that ATP hydrolysis only takes place in site 1 during the catalytic dwell that occurs after the 80° sub-step of γ rotation is complete [14,15,93,94,95,96]. Hence, in ATP hydrolysis, i.e., the bond cleavage step in site 1 cannot be proposed as a driving force for the 80° rotational sub-step in F_1_-ATPase.

Models based on structural considerations [2] or based on electrostatic or mechanical considerations have also been proposed for both ATP hydrolysis and ATP synthesis [29,31,107,108,112]. A model based on analysis of the fluoroaluminate (ADPAlF_4_^−^)_2_ F_1_ X-ray structure [2] that also proposed that both binding and hydrolysis steps drive rotation is beset with the same problem discussed above. Moreover, as shown by us previously [31], the model is effectively bisite: it appears trisite only due to the way it is labeled [2]. Models that invoke ATP binding to site 3 as solely or primarily responsible for driving rotation [7,93,94,107,108] are also problematic for the reasons spelled out above. Other models (e.g., see ref. [82]) propose that catalysis (i.e., the hydrolysis event) occurs during the tight to open transition of a catalytic site, which contradicts structural evidence that suggests that opening of the β-subunit occurs after ATP hydrolysis [2]. Several other difficulties with the proposed models of ATP synthesis and hydrolysis have been discussed previously [31,33,34,97]. 

This is not to say that the experimental data collected by the above single-molecule works [14,15,93,94,95,96] are incorrect. They are correct under the conditions, as are the structural data and the consolidated biochemical and biophysical information in the field. What is required is a molecular mechanism of ATP synthesis/hydrolysis by the ATP synthase/F_1_-ATPase that is consistent with the entire body of available experimental information.

Attempts to revisit ATP synthesis/hydrolysis catalysis by ATP synthase have been made [30,31,38,57,92,113]. In a recent comprehensive work, Frasch re-analyzed the molecular mechanisms of both ATP synthesis and hydrolysis based on gold nanorod probes and single-molecule microscopy measurements using single-photon counting avalanche photodiodes [38]. Their model of hydrolysis by F_1_ proposes that Pi release from β_DP_ and ATP binding to β_E_ drives the two phases of the power stroke (Figure 10 in ref. [38]). The model has similar shortcomings as the models discussed above, in that site 3 is open and distorted and cannot support the phase 2 power stroke, as postulated [38]. In fact, all models in which MgADP release precedes Pi release do not satisfy trisite catalysis [14,38,96] because, in these models, rotation does not occur with all three catalytic sites occupied by bound MgATP or bound MgADP.

A true trisite model of ATP hydrolysis that does not have the above shortcomings is one proposed by Nath in Reference [31]. However, even this model is not fully correct if we accept Senior and colleagues’ finding using their optical probes in fluorescence experiments that during steady state V_max_ catalysis, the predominant long-lived enzyme species contains two bound MgADP and one bound MgATP [34,105]. Such a state is considered to be one that occurs after an 80–90° rotation from the ATP binding state at 0° [105]. In other words, at high [ATP], product Pi release is rate-limiting. If the model of Reference [31] is revised to include the fact that the ATP molecule in site 2 that binds upon ligand displacement (ADP–ATP exchange in site 2) hydrolyzes and releases Pi from that site [92,113], then the revised model (Figure 10; see panels 4 and 5) is consistent both with the biochemical observations [105] and with the results of the oxygen exchange experiments in this work. A 2023 paper describes in considerable detail the various states/angular positions along the catalytic pathway of F_1_-ATPase [92] and their correspondence with snapshots represented by relevant high-resolution X-ray and cryo-EM structures according to Nath’s torsional mechanism of energy transduction and ATP synthesis/hydrolysis. The biological significance and implications of the work [92] have been discussed in a Perspective Article by Wray [113].

To sum up, a wealth of novel mechanistic insights, along with a detailed molecular model that also elucidates and explains the how and why in ATP hydrolysis mechanism by F_1_-ATPase, has been formulated based on stochastic kinetic analysis of the oxygen exchange reactions. Previously, a view had been expressed that the ^18^O exchange technique can only offer *qualitative* information on the mechanism [34]. The present work on ATP hydrolysis by F_1_-ATPase and our previous work on ATP synthesis by the F_O_F_1_-ATP synthase [57] has falsified this view. It has shown definitively that given the right probabilistic approach to the analysis of oxygen exchange data (Section 3), unique insights (Section 5.3, Section 5.4, Section 5.5 and Section 5.6) and unprecedented information (Section 5.7, Section 5.8 and Section 5.9) on molecular mechanisms can indeed be obtained. These detailed insights had proved very difficult to obtain previously by other biochemical and biophysical techniques.

## 6. Conclusions

A quantitative analysis of the intermediate Pi–HOH exchange accompanying ATP hydrolysis by F_1_-ATPase over a wide substrate ATP concentration range—from sub-micromolar to millimolar—has been carried out. The kinetic data covered the full range of oxygen exchange that can be mediated by the F_1_-ATPase. Both the overall extent of oxygen exchange as well as the distribution of all [^18^O]Pi isotopomer species—containing 0, 1, 2, and 3 ^18^O atoms in the Pi released from β-catalytic sites into the medium—were quantified over the 50,000-fold medium ATP range. The analysis of oxygen exchange was performed by application of a stochastic kinetic theory specifically developed for that purpose. The following conclusions were reached in this study based on oxygen exchange methodology:

1. A single site of entry of water oxygen—a fundamental limitation of the model of oxygen exchange proposed by Boyer’s binding change mechanism of ATP synthesis/hydrolysis—is inadequate to predict even gross features of the exchange process.
2. Multiple sites of water entry, as proposed by Nath’s torsional mechanism of ATP synthesis/hydrolysis since 2003 [32], are essential to explain exchange data.
3. A *constant* value for the apparent rate constant of oxygen exchange (k) of 10.5 ± 0.1 s^−1^ is found over five decades of ATP concentration during steady-state ATP hydrolysis by mitochondrial F_1_-ATPase.
4. Finding 3 contradicts a fundamental tenet of the binding change mechanism that postulates that medium ATP concentration markedly modifies values of the rate constant(s) governing the oxygen exchange of Pi bound at the catalytic site prior to its release [58,59,60] and neglects consideration of a timescale for the exchange.
5. The results are consistent with the torsional mechanism that considers the time available for exchange, t, by the bound nucleotide/Pi in the catalytic site as the appropriate timescale and postulates that this parameter quantitatively determined the observed extent of the oxygen exchange when multiplied by an essentially constant apparent rate constant for the exchange process, k. According to this theory, changes in the medium substrate concentration alter t, which then is responsible for the modulation of the extent and rate of oxygen exchange.
6. **Ligand permutation** is the fundamental cause for the occurrence of the intermediate Pi–HOH exchange (during ATP hydrolysis) (this work) and the intermediate ATP–HOH oxygen exchange (during ATP synthesis) [57], as opposed to multiple spontaneous reversals of ATP synthesis/hydrolysis in a single catalytic site [60].
7. The exchanges occur mechanistically because the enzyme catalytic site lacks absolute spatial selectivity for the oxygen ligands of a phosphorus intermediate that it accepts and binds as substrate. Hence, the oxygen ligands readily exchange, i.e., *permute* their positions about the central phosphorus atom.
8. Three catalytic sites contribute to oxygen exchange at short times (high [ATP]) that, however, reduce to two sites mediating intermediate Pi–HOH exchange at long times (low [ATP]), as revealed by the detailed experimental distribution of [^18^O]Pi isotopomer species found in our work and its stochastic kinetic analysis.
9. The finding 8 imposes strong constraints on possible molecular mechanisms of ATP hydrolysis by F_1_-ATPase.
10. None of the existing mechanisms, in their current form, fully explain the experimental observations in 8 and present a solution to the mechanistic conundrum.
11. The concept of **ligand displacement** was proposed in 2008 for **bisite activation** of the F_1_-ATPase by ATP binding at a second catalytic site by the torsional mechanism and the unified theory [31], with the additional new element that the enzyme needs to also hydrolyze the bound ATP (that had exchanged with bound ADP in site 2 by **substitution chemistry**) to ADP.Pi, and subsequently unbinding and releasing the Pi from the site, presently offers the only way to resolve the mechanistic conundrum [92,113].
12. **β_TP_** in the Leslie–Walker structures [1,2] (PDBID: 1BMF, 1H8E) is definitively identified as **site 2** on the basis of our oxygen exchange data.
13. Pi release **precedes** ADP release in the catalytic cycle of steady-state ATP hydrolysis by F_1_-ATPase.
14. The elementary chemical processes and the angular position at which they occur during ATP hydrolysis by the complete F_1_-ATPase that are consistent with the results of the oxygen exchange experiments in this work are ATP binding—**0°**, ATP bond cleavage—**200°**, Pi release—**200°**, and ADP release [92]—**240°**.
15. F_1_-ATPase performs its function of steady-state V_max_ hydrolysis using a **trisite** mechanism, i.e., by a mode of operation in which rotation and catalysis occur with 3-site filling of β-catalytic sites by the Mg-nucleotides MgATP or MgADP. This finding contradicts **bisite** models of steady-state catalysis by ATP synthase/F_1_-ATPase proposed within Boyer’s binding change mechanism [60] but is fully consistent with Nath’s torsional mechanism of energy transduction and ATP synthesis [31,33] and hydrolysis [31,92].

These results and conclusions need to be taken into account in future work on ATP synthesis/hydrolysis by the F_O_F_1_/F_1_-ATPase [113]. 

## Data Availability

The data are contained within the article or the Supplementary Materials.

## Supplementary Materials

The following supporting information can be downloaded at https://www.mdpi.com/article/10.3390/biom13111596/s1, Computer program for the analysis of kinetic data.
