# Supplementary material for: Elucidating Events within the Black Box of Enzyme Catalysis in Energy Metabolism: Insights into the Molecular Mechanism of ATP Hydrolysis by F1-ATPase
Source: Biomolecules. 2023 Oct 30;13(11):1596. doi: 10.3390/biom13111596 (PMC10669602; doi:10.3390/biom13111596)
Supplement: Supplementary file 1 [file biomolecules-13-01596-s001.zip › biomolecules-2619151-supplementary.pdf]

Supplementary Information

Elucidating Events within the Black Box of Enzyme Catalysis in Energy Metabolism: Insights into the Molecular Mechanism of ATP Hydrolysis by F_1-ATPase

Sunil Nath[1,2]*

[1]Department of Biochemical Engineering and Biotechnology, Indian Institute of Technology Delhi, Hauz Khas, New Delhi 110016, India

[2]Visiting Professor, Institute of Molecular Psychiatry, Rheinische-Friedrichs-Wilhelm Universität Bonn, D–53127, Bonn, Germany

*E-mail: sunath@iitd.ac.in; sunil_nath_iit@yahoo.com

C^{++} COMPUTER PROGRAMS (PLATE-1 and PLATE-2)

PLATE-1

THE PROGRAM USING THE EQUATIONS DERIVED FOR DIFFERENT RATE CONSTANTS FOR DIFFERENT OXYGEN TRANSFER.

```
#include<iostream.h>
#include<math.h>
#include<iomanip.h>

void main()
{
double p00,t,dt,x,v,w,y,z;
v=0;w=0;y=0;z=0;
double a1,a2,a3,a4;
double b1,b2,b3,b4;
double p0,p1,p2,p3,p4;
int i,n;

cout<<"please input the value of Po"<<endl;
cin>>p00;
cout<<"input the increment dt"<<endl;
cin>>dt;
cout<<"input valueRate Constant : "<<endl;
cin>>k;
cout<<"input the no. of iterations"<<endl;
cin>>n;
t=0;
if (p00>x) {cout<<"t"<<"      ,"<<"p0"<<"     ,"<<"p1"<<"     ,"<<"p2"<<"     ,"<<"p3"<<"
        ,"<<"p4"<<endl;
        for(i=0;i<n;++i){
                        t=t+dt;
                        p0=p00-(a1*t);
                        if ((p0<0)||(p0==0)) {p0=0;if (v==0) {v =t;}};
                        if (p0 != 0) {p1=(a1-a2)*t;} else {p1=((a1-a2)*v)-a2*(t-v);};
                        if ((p1<0)||(p1==0)) {p1=0;if (w==0) {w =t;}};
                        if (p1 != 0) {p2=(a2-a3)*t;} else {p2=((a2-a3)*w)-a3*(t-w);};
                        if ((p2<0)||(p2==0)) {p2=0;if (y==0) {y =t;}};
                        if (p2!=0) {p3=(a3-a4)*t;} else {p3=((a3-a4)*y)-a4*(t-y);};
                        if ((p3<0)||(p3==0)) {p3=0;if (z==0) {z = t;}};
                        if (p3!=0) {p4=a4*t;} else {p4=z*a4;};
                        cout<<t<<"      ,"<<p0<<"      ,"<<p1<<"      ,"<<p2<<"
        ,"<<p3<<"       ,"<<p4<<endl;
                        if (p4>.391) {break;};
                        }
        }
else {cout<<"  ,"<<"t"<<"      ,"<<"p0"<<"     ,"<<"p1"<<"     ,"<<"p2"<<"     ,"<<"p3"<<"
        ,"<<"p4"<<endl;
                for(i=0;i<n;++i){
                        t=t+dt;
                        p0=p00*(exp(-b1*t));
                        p1=((b1*p00)/(b2-b1))*((exp(-b1*t)-exp(-b2*t)));
                        p2=(((b1*b2*p00)/((b2-b1)*(b3-b2)))*exp(-
(b1*t)))+(((b1*b2*p00)/((b1-b2)*(b3-b2)))*exp(-(b2*t)))+(((b1*b2*p00)/((b1-b3)*(b2-
b3)))*exp(-(b3*t)));
                        if ((p2<0)||(p2>.391)) { p2=0;};
                        p3=(((b1*b2*b3*p00)/((b2-b1)*(b3-b2)*(b4-b1)))*exp(-
(b1*t)))+(((b1*b2*b3*p00)/((b1-b2)*(b3-b2)*(b4-b2)))*exp(-
(b2*t)))+(((b1*b2*b3*p00)/((b1-b3)*(b2-b3)*(b4-b3)))*exp(-(b3*t)))-
(((b1*b2*b3*p00)/((b4-b1)*(b4-b2)*(b4-b3)))*exp(-(b4*t)));
                        if ((p3<0)||(p3>.391)) { p3=0;};
                        p4=p00-p0-p1-p2-p3;
                        // if (p0<0) {p0=0;};if (p1<0) {p1=0;};if (p2<0) {p2=0;};if
(p3<0) {p3=0;};if (p4<0) {p4=0;};
                        cout<<t<<"      ,"<<p0<<"      ,"<<p1<<"      ,"<<p2<<"
        ,"<<p3<<"       ,"<<p4<<endl;
                        if (p4 > .391) { break;};
                        }
        }
}
```

The difficulty in understanding of the program is due to the complex nature of the equations used.

PLATE-2

THE PROGRAM USING THE EQUATIONS USING SAME RATE CONSTANT FOR ALL THE STEPS IN THE OXYGEN TRANSFER TO THE PHOSPHATE MOLEULE.

```
#include<iostream.h>
#include<math.h>
#include<iomanip.h>

void main()
{
double p00,p0,p1,p2,p3;
int i,n;
float dt,t,k;

cout<<"please input the value of Po"<<endl;
cin>>p00;
cout<<"input the increment dt"<<endl;
cin>>dt;
cout<<"input Rate Constant : "<<endl;
cin>>k;
cout<<"input the no. of iterations"<<endl;
cin>>n;
t=0;
cout<<"t"<<"          ,"<<"p0"<<"          ,"<<"p1"<<"
     ,"<<"p2"<<"          ,"<<"p3"<<endl;
     for(i=0;i<n;++i){
               t=t+dt;
               p0=p00*exp(-(k*t));
               p1=k*(p00*exp(-(k*t))*t);
               p2=(k*k*t*t*p00*exp(-(k*t))/2);
               p3=p00-p0-p1-p2;
               cout<<t<<"         ,"<<p0<<"
     ,"<<p1<<"      ,"<<p2<<"      ,"<<p3<<endl;
               }

}
```
